# Establish operating conditions for optimal output characteristics in reactivity controlled combustion engine

**DOI:** 10.1038/s41598-025-00497-2

**Published:** 2025-05-16

**Authors:** Sinnappadass Muniyappan, Ravi Krishnaiah

**Affiliations:** School of Mechanical Engineering, VIT University, Vellore, 632014 Tamilnadu India

**Keywords:** Reactivity controlled compression ignition (RCCI), DI Mahua biodiesel-diesel as high reactive fuel (HRF), Port fuelled ethanol injection as low reactive fuel (LRF), Simultaneous reduction of smoke and NOx, RSM for engine characteristics optimization., Climate sciences, Environmental sciences, Environmental social sciences, Energy science and technology, Engineering

## Abstract

This work aims to determine the optimal engine operating conditions for balanced combustion, performance and emissions characteristics with considerable reduction in smoke and nitrogen oxides (NOx). This work examined direct injected (DI) 30% mahua biodiesel-diesel as high reactive fuel (HRF) and port fuel injected (PFI) ethanol as low reactive fuel (LRF) in reactivity controlled compression ignition (RCCI) combustion at different engine loads and ethanol energy shares (EES) (0, 10, 15, 20, 25, and 30%). The RCCI engine was able to access low temperature combustion (LTC) with improved brake thermal efficiency (BTE), lower smoke and NOx with a trade-off in carbon monoxide (CO) and hydrocarbon (HC). The duty conditions of modern engines require single optimal operating condition to suit applications such as hybrid powertrain, generators and irrigation pumps. Using response surface methodology (RSM) it was established that 28.43% EES at 83.4% engine load resulted in optimal output responses for their due weightages assigned. This was validated by experimental results. In RCCI mode BTE of 32.54%, brake specific energy consumption (BSEC) of 10.79 MJ/kWh were realized. Also, smoke and NOx were reduced by 34.8% and 29.3%, with a compromise in CO and HC increase by 36.4% and 34.9% compared to DI mode. All the engine output parameters reported were within acceptable range. HC and CO can be mitigated with conventional catalytic convertors.

## Introduction

Diesel engines are used as power sources in transportation, agriculture, manufacturing, and construction due to their robustness and greater fuel conversion efficiency compared to petrol engines. Moreover, in the last two decades, the use of high-pressure electronically controlled fuel injection systems alongside the implementation of modern aftertreatment systems resulted in diesel engines’ significant emissions of NOx and soot reduction. Moreover, engine manufacturers and researchers are creating alternative combustion models using new fuels for diesel engines governed by diffusion combustion in response to stricter exhaust pollution regulations^1^. The inclusion of a high octane number (ON) fuel prior to induction and compression, followed by ignition via the injected diesel fuel, has demonstrated an improvement in premixture homogeneity in the advanced engine modification approach^2^. Compression ignition(CI) engines produce exhaust emissions that are heavily influenced by the fuel type used. Changing fuels may therefore lower these pollutants. In an effort to reduce exhaust emissions, several research have suggested mixing diesel with other fuels that include oxygen(O_2_) or infusing these fuels straight into the combustion chamber^3^. The depletion of conventional fuels, increasing prices, and growing greenhouse gas emissions (GHG) have all boosted demand for environmentally acceptable, cost-effective, renewable energy that decreases GHG. Thus, fossil fuels must be replaced with other fuels. To solve existing issues and prevent climate change, diesel must be replaced with a sustainable fuel. In addition, biofuels like biodiesel, which has become a viable fossil fuel alternative, enhance combustion and decrease GHG^4^. Biodiesel is an environmentally friendly, non-combustible, non-toxic, renewable, and biodegradable alternative energy source. The growing countries with huge population need to get its biofuel from a source that can’t be eaten, like the Mahua tree, which is also called madhuca indica^5^. One of the most popular vegetable oils, mahua oil, offers diesel like qualities, such as a greater cetane number (CN) and a higher calorific value (CV). As a result, it might be used as a substitute fuel for CI engines. Panigrahi et al. investigated the effects of a diesel fuel additive that included mahua biodiesel on performance, emissions, and health. On the other hand, issues such as higher brake specific fuel consumption (BSFC) and higher NOx pollution make them less useful in CI engines^6,7^. In this regard, researchers have investigated many combustion modes which makes use of renewable, low-carbon fuels that are less harmful to the environment than fossil fuels. These research help decrease the demand for fossil fuels by making internal combustion engines (ICE) more sustainable and ecologically friendly. Researchers have recently tested using biodiesel and used bio oils^8,9^. The most promising, clean, and alternative diesel fuel is biodiesel. Alcohol transesterification of vegetable oils or animal fats produces biodiesel without aromatics or sulphur. It also includes 10–15% O_2_ content by weight. It may be used straight or blended with diesel in CI engines. Low volatility, high density, high viscosity, and high pour point are drawbacks of biodiesel^10^. Biodiesel fuel has a greater CN and O_2_ concentration than diesel, hence it helps minimise soot emission when utilised as an ignition source for premixed fuel in dual fuel (DF) combustion^11^.

Fuel physical qualities are crucial to diesel engine atomisation. For instance, biodiesel viscosity improves fuel droplet size distribution, fuel injection atomisation, and mixture consistency. Alcohol enhances biodiesel blend qualities by lowering density and viscosity. Due to their high O_2_ concentration, alcohols may minimise exhaust emissions and increase engine combustion in diesel and biodiesel fuel^12^. A key indicator of fuel ignition quality and ignition delay (ID) duration is the CN. CN rises according to carbon chain length. Common diesel engines need a CN between 45 and 60, and when it drops below 38, ID rises fast. Ethanol, methanol, and butanol have much lower CN (8, 3, and 25) than diesel fuel, leading in longer ID. The O_2_-rich alcohols increase premixed and diffusion combustion phases, but their lower heating values and CNs, miscibility and stability issues, poor auto-ignition, and improper lubricating abilities restrict their usage as diesel engine fuel. A number of experiments have examined engine performance and exhaust emissions utilising alcohol fuels (ethanol, methanol, and butanol) combined with diesel^13^. Due to its renewable nature and diesel fuel miscibility, ethanol is the most widely employed alcohol in fuel research. Due to its high O_2_ content, ethanol enhances combustion and decreases CO and smoke. Ethanol is a sustainable fuel made from biomass like maize, sugar beets, sugar cane, sweet sorghum, barley, cassava, and molasses via alcoholic fermentation. Produce ethanol using agricultural wastes such raw materials, scrap wood, and straw^14^. Due to its unique features (increased O_2_ content, latent heat of vapourization (LHV), etc.) and renewable fuel status, methanol is being extensively studied. DF combustion can minimise NOx and soot suffered by traditional diesel engines^15^. Compared to diesel at full load 20% karanja biodiesel with 10% ethanol blend reduced BTE by 2%, BSFC by 3%, exhaust temperature by 3%, CO_2_ by 0.86%, HC by 12 ppm, CO and NOx by 0.029% and 8%, respectively^16^. Karin et al. investigated the 7% palm oil biodiesel with 20% ethanol blend, which resulted in reduced cylinder pressure and increased HRR, leading to an increase in BTE and BSFC^17^. According to a study, the higher LHV associated with lower alcohol results in increased heat absorption during combustion, lowering the combustion temperature and possibly impacting the engine’s cold start qualities. Furthermore, several studies have shown that adding ethanol into diesel fuel resulted in lesser NOx and soot^18^. Ethanol high ON fuel that use a LTC method^19^. Ethanol is frequently employed in the production of diesel-based blends due to its low CN, high self-ignition temperatures, and high LHV. Biodiesel is an effective surfactant for diesel mixtures because phase separation occurs when ethanol combines with diesel at temperatures below 10˚C^20^.

Advanced combustion techniques including homogeneous charge compression ignition (HCCI), premixed charge compression ignition (PCCI), and RCCI can be deployed without equipment or engine changes, have been extensively studied for emission reduction. To avoid high-temperature zones in the combustion chamber, diffusion-phase combustion, and the production of NOx and soot, combustion is regulated by premixed charge and fuel reactivity^21^. Benajes et al. found that switching to conventional mode is necessary to reach maximum load without excessive pressure increase^22^. In order to reduce NOx and soot, premixed combustion must be reduced due to high pressure rise rate with load. To broaden the, load range and lower excessive HC and CO, additional extensive investigations are required in PFI strategy^23^. The LRF to HRF ratio determines the mixture’s global reactivity, whereas the ID stratification controls combustion time, combustion duration (CD) and phasing in the RCCI mode. HRF-air mixture spots initiate combustion, which progresses from high-to-low reactivity for regulated sequential ignition. It outperforms other LTC combustion control techniques. For a broad range of speeds and loads, RCCI combustion mode produces low soot and NOx and improves engine efficiency. The initial injection boosts in-cylinder reactivity. The second injection enhances mixing and ignites, reducing soot. By adjusting global charge reactivity and direct injections, combustion phasing and heat release rate (HRR) can be entirely controlled^24^. In a study, diesel is injected directly and gasoline and biogas into ports. Compared to gasoline-diesel RCCI mode combustion, biogas-diesel DFC mode combustion required more time for ID, and increasing the amount of PFI resulted in longer ID. Further, although NOx and soot decreased dramatically, HC and CO increased while using DF mode^25^. Telli et al. investigated RCCI combustion using diesel and hydrous ethanol. The results indicated that when hydrous ethanol constituted up to 80% of total fuel energy, significant BTE and reduced NOx and soot. At low load, NOx and soot were reduced by 79% and 50%, respectively^26^. In comparison to the traditional mode, the RCCI reduced smoke, NOx, and BTE^27^. The RCCI study using n-pentanol and cottonseed biodiesel discovered a 7% improvement in BTE at a 20% pentanol energy ratio^27^. Only a small number of research looked at using ethanol as an additional fuel in the DF mode^28^. Ethanol/diesel and ethanol/biodiesel HCCI engines had lower NOx and smoke than diesel engines with ID increased, CD decreased, in-cylinder pressure and temperature decreased. HCCI engines produce far more HC and CO than diesel engines^29,30^. Hydrotreated vegetable oil (HVO) is used in diesel-like fuels to test its high CN. HVO-ethanol had enabled the largest ethanol substitution(84%)^31^.

For the research of multivariate analysis, many non-linear techniques like design of expert (DoE), fuzzy logics, artificial neural networks, Taguchi, genetic algorithm, and RSM are necessary to reduce the complexity and improve the analysis^32^. RSM approach makes use of analysis of variance (ANOVA) and factorial-based methodologies to get proper experimental design and analysis. The quadratic models produced using RSM provide consistent and reliable prediction models for many responses^33^. The optimisation of process parameters in a diesel engine was examined using RSM for predicting and optimizing various alcohols (DEE, Ethanol) and biodiesel blends. Engine parameters, including blend ratio and engine load, were optimised to reduce BSFC, HC, CO, and NOx while enhancing BTE and peak CP^34^. Most of the studies made use of trials in which one variable was changed at time. Based on the described research complexities, concluded that RSM is an appropriate tool establish ideal operating conditions considering at numerous output responses more precisely. These property outlines recommend using CI frameworks with highly enhance fuel–air mixture homogeneity, which speeds combustion at lower peak temperatures and provides lean, clean combustion for lower emissions and fuel efficiency. Despite government awareness and marketing initiatives and its status as a fuel blend, ethanol is valuable because of its significance to evolving compression ignition-LTC transportation models now and in the future. Therefore, ethanol has been investigated in all LTC domain components, and studies have indicated that biodiesel-based LTC solutions are the best option. From the literature, it is clearly understood that use of alcohol-based fuel additives with diesel is a better option to reduce the NOx and soot emissions. To overcome challenges in dual fuel HCCI and RCCI combustion engine, many researchers used alcohols for part-load or specific load conditions. Few researchers have studied varying load conditions with ethanol as LRF. In this work, varying load conditions are investigated by supplying different energy share of ethanol with the help of fuel injector which is connected to the inlet manifold. The objectives of the present work is to reduce NOx and smoke up to zero levels as well as to increase the fuel economy in dual fuel mode RCCI combustion mode. Also using RSM prediction and optimization we have determined appropriate engine input parameters for optimized engine output parameters by assigning due weightage to each of the output parameter. The determined input factors will be ideal settings for charging the batteries in hybrid power, generator and irrigation pump installations.

## Experimental set-up

**Fig. 1:**
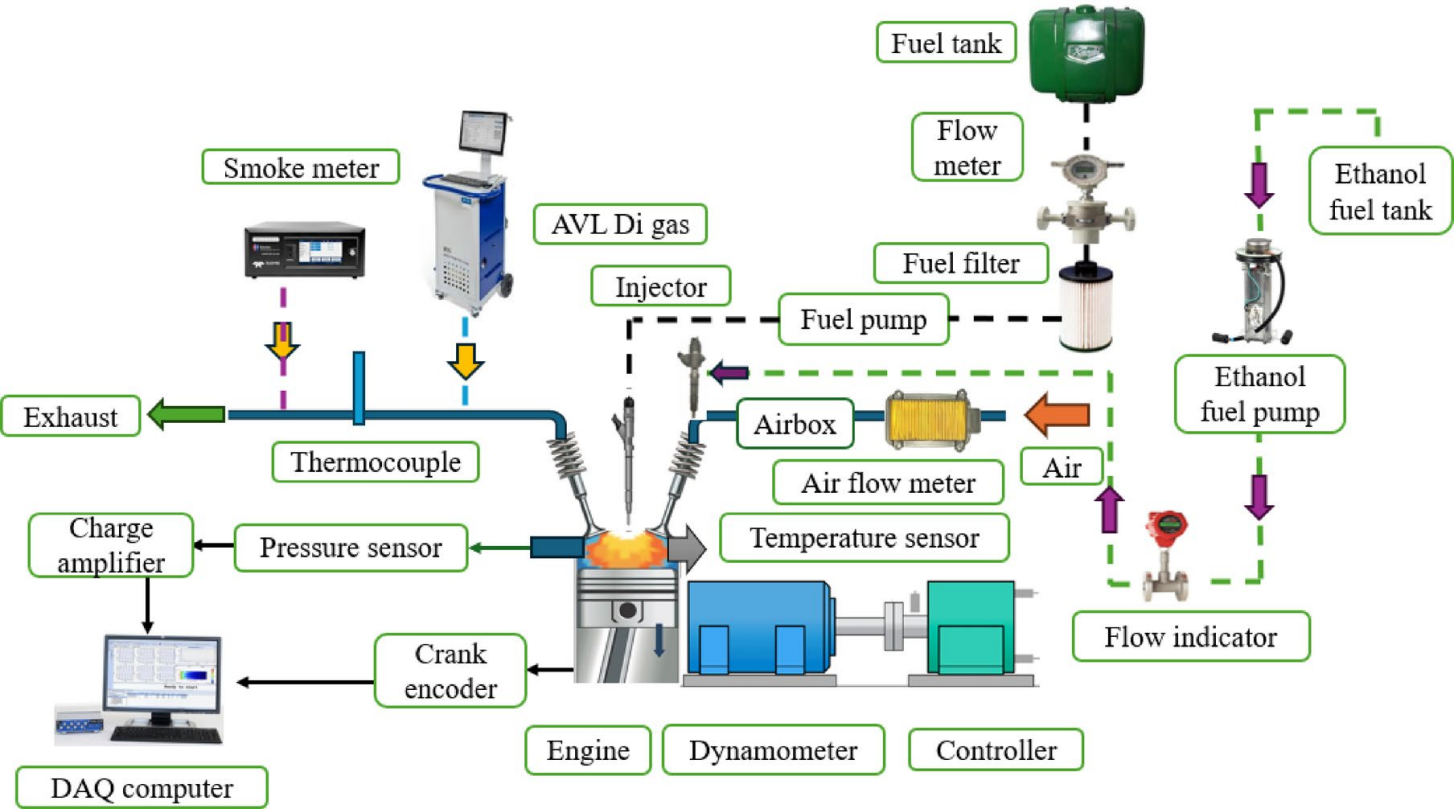
Experimental layout.

Experimental studies performed on the single cylinder direct injection diesel engine as shown in Fig. 1. The engine’s technical specs are shown in Table 1. Before adding mahua bio-diesel, the engine is warmed up with pure diesel. After determining the steady-state condition, the engine was run at 1500 rpm with a 100% load for the length of the test. The excess diesel fuel in the pipe, pump, and valves was removed prior to the introduction of the test fuels. For tests, the engine is operated on bio-diesel till the diesel is finished. The repeatability of the dynamometer was tested using a hot setup with calibration being performed pre- and post-testing of the engine. The test setup was run until the engine reached operating temperature (monitoring the engine’s oil temperature), after which the first calibration was performed. An eddy current dynamometer regulates load, and the data collecting and processing system monitors cylinder pressure, combustion parameters, and engine efficiency. A mechanically operated injector feeds HRF fuel into the cylinder at a timing of 23˚CA bTDC under all engine conditions. To function effectively, the RCCI needed at least two fuel tanks. Each fuel is thus allocated to a separate tank. A commercial PF injector was used to feed the ethanol (LRF) into the intake port. PF injectors are mounted upstream of intake manifolds to direct fuel spray near the intake valve. Fuel pump and solenoid fuel injector are calibrated for fuel injection amount and pulse width. A separate fuel injection controller controls and maintains 2 bar PF fuel pressure. Micro-controller, driver, potentiometer, and power supply make up port fuel injection controller. Microcontroller receives cam sensor input and calculates fuel injection pulse. Electrical pulse operates solenoid fuel injector, and pulse length determines fuel injection duration. Fuel injection duration is regulated by potentiometer, which controls electric pulse width. Data acquisition system (DAQ) was also responsible for measuring cylinder head temperature, intake air pressure, engine speed, and other engine data. The rate of intake airflow as measured by a thermal mass flow meter type. After warmup operation, RCCI mode is achieved by injecting ethanol into the intake manifold. Increased EES was achieved by decreasing diesel-biodiesel blend mass per engine cycle and increasing ethanol mass by lengthening ethanol injection time. These injection durations were calculated using diesel injection mass per injection. Fixed ethanol injection timing at intake TDC allowed a lengthy period for mixture preparation in the intake manifold. In a temperature range of 5–45˚C and a relative humidity of less than 95%, the CO, HC, and NOx were measured by the AVL DIGAS 444 multi-gas analyser that was attached to the exhaust pipe. A computer-based data collection system monitored the whole system, and the gas analyser required 7 min to warm up. The measurement of smoke is done using a 437-C type smoke meter. ASTM standards were used to test and measure all attributes as shown in Table 2. Study observations were collected three times and averaged. Experiments using ethanol energy portions at 20, 40, 60, 80, and 100% loads assessed the test engine’s performance and emissions in the RCCI mode. The ethanol injection time was restricted to the injector opening’s minimum pulse duration. As the fraction of ethanol injection into the cylinder increased, the amount of M30 blend reduced in order to keep the engine running at a constant 1500 rpm at the test load. When the engine was steady, data was collected at each step of the test. Finally, in the additional part, the necessary calculations for interpreting the data generated by this trial are described.

**Table 1:** Technical specifications of the test engine.

Parameters	Specification
Brand	Kirloskar engines
Configuration	Single cylinder
Displacement	Four stroke, Water cooled
Bore	87.5 mm
Stroke	110 mm
Max speed	1500 rpm
Rated power	3.5 kW
CR range	17:1
Injection angle	23˚bTDC @ M30 blend
Injection pressure	300 bar
Loading system	Eddy current dynamo meter
Port fuel injection	Ethanol
Cooling system	Water cooling

**Table 2:** Properties of diesel, biodiesel, and ethanol^35,36^.

Properties	Diesel D100	M100	M30	Ethanol
Chemical formulae	**-**	**-**	-	C_2_H_6_O
Viscosity (mm^2^/s)	1.96	3.98	2.92	1.2
Density (kg/m^3^)	830	880	852	780
Cetane Index	52.4	42	51	8
Flash point (˚C)	42	157	-	12.7
Fire point (˚C)	68	185	-	13.8
Calorific value (kJ/kg)	43,500	39,600	41,600	28,320
Auto ignition temperature (˚C)	230	-	-	365
Latent heat of vapourization (kJ/kg)	250	350	-	850
Oxygen content (%)	0	15.5	-	34.8

### Uncertainty analysis

The uncertainty analysis was used to evaluate the errors arising from the measurement of various parameters, so ensuring the reliability and accuracy of the collected data as shown in Table 3. This strategy is crucial for analysing changes and their impact on results. Uncertainty analysis is a conventional method in engine parameters and operations that offers a systematic way to identifying, evaluating, and mitigating sources of inaccuracy. This leads to more dependable and efficient mechanical outputs by improving measurement accuracy as well as optimising and certifying engine performance. The root mean square error (RMSE) method is used to determine the engine parameter uncertainty in equ(1).

1 $$Total~uncertainty\% ~~ = ~\sqrt {\left( {speed} \right)^{2} + \left( {torque} \right)^{2} + \left( {smoke} \right)^{2} ~ + \left( {Nox} \right)^{2} + \left( {HC} \right)^{2} + ~\left( {CO} \right)^{2} }$$

$$Total~uncertainty~\% ~ = ~\sqrt {\left( {1.42} \right)^{2} + \left( {0.10} \right) + \left( {1.29} \right)^{2} ~ + \left( {4.84} \right)^{2} + \left( {2.56} \right)^{2} + ~\left( {0.0135} \right)^{2} } ~~ = {\text{~}}3.31{\text{\% ~}}$$

**Table 3:** Uncertainty analysis.

Parameters	Measuring range	Uncertainty
Engine speed	> 2000 rpm	± 1.42
Torque	10.5 Nm	± 0.10
CO	0–10%	± 0.0135
HC	0–2000 ppm	± 2.56
NOx	0–5000 ppm	± 4.84
Smoke	0–100 HSU	± 1.29

### Ethanol premixing ratios

Changes in electronic fuel injection pulse width modified the energy fraction of ethanol from 0, 10, 15, 20, 25, and 30%. The ethanol premixing proportion is the % of ethanol energy in the overall fuel energy used by the engine for combustion in equ(2). The flow rates for biodiesel and ethanol are M_biodiesel_ and M_ethanol_, respectively. The CV of biodiesel is 41.6 MJ/kg, whereas that of ethanol is 28.3 MJ/kg. Table 4 demonstrates the mass flow rate of ethanol and biodiesel blend for various EES and load conditions. Table 5 illustrate the composition of mahua oil.

2 $${\text{BSEC }}\left( {{\text{MJ}}/{\text{kW}} - {\text{hr}}} \right) = ~\frac{{{\text{Methanol~}} \times {\text{CVethanol}}}}{{\left( {{\text{Methanol~}} \times {\text{CVethanol}}} \right) + \left( {{\text{Mbiodiesel~}} \times {\text{CVbiodiesel}}} \right)}} \times 100$$

3 $$~{\text{Duty cycle }} = \frac{{{\text{Time~duration~of~PFI~turned~on}}/{\text{cycle}}}}{{{\text{Total~duration}}/{\text{cycle}})}} \times 100$$

The 4-stroke CI engine at 1500 rpm has a total duration/cycle of 720˚ CA (80 ms). The maximum injection time/cycle is 220˚ CA, which is between the earliest feasible opening (20˚ bBDC) and the latest closing of the injector (20˚ aTDC), resulting in a maximum duty cycle of 30.5% with 24.4 ms of injection duration each cycle. The mass of fuel per cycle can be adjusted by changing the duty cycle in equ(3)^37^.

**Table 4:** Mass flow rate calculation for various EES at different loads.

Load	20%		40%		60%		80%		100%	
Mass flow rate (kg/hr)										
EES(%)	Ethanol	M30	Ethanol	M30	Ethanol	M30	Ethanol	M30	Ethanol	M30
0	0	0.61	0	0.78	0	0.86	0	0.88	0	1.35
10	0.07	0.44	0.11	0.72	0.13	0.8	0.13	0.8	0.19	1.19
15	0.10	0.4	0.17	0.68	0.18	0.71	0.20	0.78	0.25	1
20	0.12	0.34	0.23	0.63	0.24	0.66	0.23	0.63	0.36	0.98
25	0.14	0.29	0.26	0.55	0.25	0.53	0.27	0.57	0.43	0.89
30	0.15	0.25	0.32	0.51	0.28	0.46	0.33	0.53	0.52	0.84

**Table 5:** Fatty acid content of Mahua oil.

S.no	Fatty content	Component name	Formula	Structure numbers	Wt.(%)
1	Palmitic acid	Hexadecanoic	C_16_H_32_0_2_	16:0	16.0–28.2
2	Stearic acid	Octadecanoic	C_18_H_36_0_2_	18:0	20.0–25.1
3	Arachidic acid	Eicosanoic	C_20_H_40_0_2_	20:0	0.0–3.3
4	Oleic acid	cis-9-Octadecenoic	C_18_H_34_0_2_	18:1	41.0–51.0
5	Linoleic acid	cis-9,cis-12-Octadecadienoic	C_18_H_32_0_2_	18:2	8.9–13.7

### Response surface methodology

Response Surface technique is a set of statistical and optimization approaches for selecting a factor that has a desired effect on the response. The goal of RSM is to utilise the effects of the experiment design to get the best possible response. The goal of RSM was to optimise processes. It establishes the relationship between the values of dependent variables and their levels and each response selected for the investigation. It is used to analyse a variety of numerical and qualitative aspects in the research to choose the best course of action. Table 6 illustrate the engine input factor^38^. After determining the dependent and independent variables, an RSM plan is created, and experimental data sets are prepared. If all conditions are identical, the created experimental sets are performed using the input parameters specified. The last step in the RSM approach is numerical optimisation. During optimisation, the desired criteria are included into the response variables, and the optimal independent variables are discovered. The RSM approach was used in this study using the DoE-12 program. Engine load and EES were utilised as RSM input values. Table 7 illustrate the RSM experimental matrix.

**Table 6:** Engine input level.

Engine Input	Code	Level		
		-1	0	1
Load (%)	A	0	50	100
Ethanol energy share (%)	B	0	15	30

**Table 7:** RSM metrics design.

Run	A: Load (%)	B: EES (%)
1	20	0
2	20	10
3	20	15
4	20	20
5	20	25
6	20	30
7	40	0
8	40	10
9	40	15
10	40	20
11	40	25
12	40	30
13	60	0
14	60	10
15	60	15
16	60	20
17	60	25
18	60	30
19	80	0
20	80	10
21	80	15
22	80	20
23	80	25
24	80	30
25	100	0
26	100	10
27	100	15
28	100	20
29	100	25
30	100	30

### Quadratic equation in engine parameters

4 $${\text{ID }}({}^{ \circ }{\text{CA}}) = {\text{13}}.{\text{229 }} - {\text{ 1}}.{\text{1}}0{\text{97 }} \times {\text{ A }} + {\text{ }}0.{\text{546 }} \times {\text{ B }} + {\text{ }}0.0{\text{2743 }} \times {\text{ AB }} + {\text{ }}0.{\text{33333 }} \times {\text{ A}}^{{\text{2}}} + {\text{ }}0.0{\text{9 }} \times {\text{ B}}^{{\text{2}}}$$

5 $${\text{CD}}({}^{ \circ }{\text{CA}}) = {\text{ 17}}.{\text{583 }} - {\text{ 4}}.{\text{4311 }} \times {\text{ A }} + {\text{ 1}}.{\text{25}}0{\text{1 }} \times {\text{ B }} - {\text{ }}0.{\text{26}}0{\text{23 }} \times {\text{ AB }} + {\text{ 1}}.{\text{3733 }} \times {\text{ A}}^{{\text{2}}} + {\text{ }}0.{\text{37136 }} \times {\text{ B}}^{{\text{2}}}$$

6 $${\text{Peak CP }}\left( {{\text{bar}}} \right) = {\text{ 57}}.{\text{632 }} + {\text{ 2}}0.{\text{124 }} \times {\text{ A }} + {\text{ 1}}.{\text{539 }} \times {\text{ B }} + {\text{ 1}}.{\text{519 }} \times {\text{ AB }} - {\text{ 1}}0.0{\text{15 }} \times {\text{ A}}^{{\text{2}}} + {\text{ 1}}.{\text{7}}0{\text{23 }} \times {\text{ B}}^{{\text{2}}} ~$$

8 $${\text{BTE }}\left( \% \right) = {\text{ 26}}.{\text{5}}0{\text{99 }} + {\text{ 8}}.{\text{6}}0{\text{689 }} \times {\text{ A }} + {\text{ 2}}.{\text{2}}0{\text{77 }} \times {\text{ B }} - {\text{ }}0.0{\text{7997 }} \times {\text{ AB }} - {\text{ 6}}.{\text{3876 }} \times {\text{ A}}^{{\text{2}}} + {\text{ }}0.{\text{2233 }} \times {\text{ B}}^{{\text{2}}} ~$$

9 $${\text{BSEC }}\left( {{\text{MJ}}/{\text{kW}} - {\text{hr}}} \right) = {\text{ 15}}.{\text{493 }} - {\text{ 4}}.{\text{788 }} \times {\text{ A }} - {\text{ 1}}.{\text{337 }} \times {\text{ B }} + {\text{ }}0.{\text{429 }} \times {\text{ AB }} + {\text{ 3}}.{\text{492 }} \times {\text{ A}}^{{\text{2}}} - 0.0{\text{418 }} \times {\text{ B}}^{{\text{2}}}$$

10 $${\text{CO }}\left( \% \right) = {\text{ }}0.{\text{1892 }} - {\text{ }}0.0{\text{86}}0{\text{ }} \times {\text{ A }} + {\text{ }}0.0{\text{21}}0{\text{ }} \times {\text{ B }} - {\text{ }}0.00{\text{3}}0{\text{5 }} \times {\text{ AB }} + {\text{ }}0.0{\text{275238 }} \times {\text{ A}}^{{\text{2}}} - {\text{ }}0.00{\text{346 }} \times {\text{ B}}^{{\text{2}}} ~$$

11 $${\text{HC }}\left( {{\text{ppm}}} \right) = {\text{ 99}}.{\text{846 }} - {\text{ 22}}.{\text{27}}0{\text{ }} \times {\text{ A }} + {\text{ 16}}.{\text{2451 }} \times {\text{ B }} - {\text{ 3}}.{\text{798}}0{\text{9 }} \times {\text{ AB }} + {\text{ 13}}.{\text{38 }} \times {\text{ A}}^{{\text{2}}} + {\text{ 1}}.0{\text{2279 }} \times {\text{ B}}^{{\text{2}}} ~$$

12 $${\text{NOx }}\left( {{\text{ppm}}} \right) = {\text{ 743}}.{\text{174 }} + {\text{ 314}}.{\text{524 }} \times {\text{ A}}~ - {\text{ 77}}.{\text{334 }} \times {\text{ B }} + {\text{ }} - {\text{29}}.{\text{453 }} \times {\text{ AB }} - {\text{ 93}}.{\text{8}}0{\text{3 }} \times {\text{ A}}^{{\text{2}}} - {\text{1}}.{\text{152 }} \times {\text{ B}}^{{\text{2}}} ~$$

13 $${\text{Smoke }}\left( {\text{N}} \right) = {\text{ 6}}.{\text{6964 }} + {\text{ 5}}.{\text{5654 }} \times {\text{ A }} - {\text{ 1}}.{\text{1683 }} \times {\text{ B }} - {\text{ }}0.{\text{34886 }} \times {\text{ AB }} + {\text{ 1}}.{\text{2476 }} \times {\text{ A}}^{{\text{2}}} ~ - 0.0{\text{8357 }} \times {\text{ B}}^{{\text{2}}} ~$$

### ANOVA analysis

This study used ANOVA to assess the significance test for engine performance and emissions parameters. A preliminary analysis was conducted on the measured responses from Table 8 to see if the variables explored had a “significant” impact on the responses. Examining the p-value and F-value is essential for validating any quadratic model. A greater F-value suggests that the model is more dependable, but a lower p-value suggests that the model is statistically significant^39^. Table 9 shows the engine input optimization criteria. The impact of various experimental factors on the results of the experiments is specified using ANOVA. The first step in an ANOVA is to estimate the impact on each component and a possibility of any possible responses. The detailed estimate of these effects’ significance is part of the second ANOVA stage. For various experimental setups, it was assumed that the experimental measures had a normal distribution with comparable variance for the F-test (a well-established test). ANOVA estimates how each independent input variable and their interaction affect output. P-value and Fisher test value (F-value) are crucial for identifying regression model suitability and significance and evaluating regression coefficient significance to create a statistically significant regression model. F-value is the ratio of mean square of group variance to error to quantify data variation relative to the mean. Regression equations with high F-values can demonstrate response variation. Each variable’s significance was established by a P-value below 0.05. Furthermore, regression model coefficient of determination R^2^, adjusted R^2^, and anticipated R^2^ were examined. Increasing model predictor variables raises R^2^ value, hence modified R^2^ is utilised to prevent this impact. As it accounts for predictor factors and shows whether the model fits experimental data. By adding helpful predictor variables, adjusted R^2^ will grow, and by adding useless ones, this will drop^40,41^.

### Optimization criteria

When ethanol is premixed into an M30 blend, input factors like as engine load and EES must be optimised to enhance BTE while reducing BSEC and exhaust emissions. The optimisation criteria, including lower and upper bounds, relevance, and weight, are determined by the aim. The weights vary from 0 to 1. A weight and importance signifies objective of the study. A weight of 0 1nd 1 and importance of 1 t0 5 scale represent the importance of each parameter (Table 10). Using the above-mentioned optimisation parameters, the RSM provided seven solutions with up to 30% EES using the desirability technique. Options with greater desire, closer to 1, are favoured.

**Table 8:** ANOVA results for developed models for different engine characteristics response.

Source	F-value	*p*-value	F-value	*p*-value	F-value	*p*-value	F-value	*p*-value	F-value	*p*-value
Parameter	ID		CD		Peak CP		HRR		BTE	
Model	537.16	< 0.0001	1195.35	< 0.0001	146.42	< 0.0001	84.28	< 0.0001	380.61	< 0.0001
A-Load (%)	2109.46	< 0.0001	5190.84	< 0.0001	639.22	< 0.0001	351.48	< 0.0001	1482.73	< 0.0001
B-EES (%)	453.92	< 0.0001	367.27	< 0.0001	3.32	0.0408	26.72	< 0.0001	86.71	< 0.0001
AB	0.5728	0.0457	7.96	0.0095	1.62	0.0216	0.04870	0.0492	0.0569	0.0413
A²	68.52	< 0.0001	179.50	< 0.0001	57.00	< 0.0001	28.15	< 0.0001	294.00	< 0.0001
B²	4.93	0.0360	12.96	0.0014	1.63	0.0244	0.0011	0.0034	0.3548	0.0470

Source	F-value	*p*-value	F-value	*p*-value	F-value	*p*-value	F-value	*p*-value	F-value	*p*-value
Parameter	BSEC		CO		HC		Smoke		NOx	
Model	153.18	< 0.0001	76.67	< 0.0001	41.08	< 0.0001	486.47	< 0.0001	208.25	< 0.0001
A-Load (%)	601.86	< 0.0001	339.94	< 0.0001	121.94	< 0.0001	2266.06	< 0.0001	949.23	< 0.0001
B-EES (%)	41.68	< 0.0001	18.03	0.0003	57.67	< 0.0001	88.76	< 0.0001	51.01	< 0.0001
AB	2.15	0.0158	0.1901	0.0068	1.58	0.0214	3.96	0.0501	3.70	0.0464
A²	115.24	< 0.0001	12.53	0.0017	15.84	0.0006	41.00	< 0.0001	30.39	< 0.0001
B²	0.0163	0.0495	0.1958	0.0421	0.0914	0.0050	0.1817	0.0437	0.0045	0.0369

**Table 9:** The regression analysis of engine output response.

ANOVA	ID (˚ A)	CD (˚ CA)	Peak CP (bar)	HRR (J/˚ CA)	BTE (%)	BSEC (MJ/kWh)	CO (%)	HC (ppm)	Smoke (*N*)	NOx (ppm)
Std. dev	0.0923	0.2349	3.04	3.20	0.8536	0.7453	0.0178	7.70	0.4465	38.98
R^**2**^	0.9911	0.9960	0.9683	0.9461	0.9875	0.9696	0.9411	0.8954	0.9902	0.9775
Adj. R^**2**^	0.9893	0.9952	0.9616	0.9349	0.9850	0.9633	0.9288	0.8736	0.9882	0.9728
Predt. R^**2**^	0.9857	0.9935	0.9460	0.9158	0.9805	0.9561	0.9087	0.8428	0.9850	0.9668

**Table 10:** Desirability function with weightage ratio of each parameters.

Name	Goal	Lower Limit	Upper Limit	Lower Weight	Upper Weight	Importance	Desirability
A: Load (%)	In range	20	100	1	1	3	1
B: EES (%)	In range	0	30	1	1	3	1
ID (˚CA)	Maximize	11.9695	15.281	1	1	1	0.67578
CD (˚CA)	Minimize	13.9064	25.2689	1	1	1	0.59619
Peak CP (bar)	Maximize	27.4929	74.1006	1	1	3	0.58344
HRR (J/˚CA)	Maximize	33.5647	70.1932	1	1	3	0.64017
BTE (%)	Maximize	9.4511	31.6074	1	1	3	0.74375
BSEC (MJ/kWh)	Minimize	12.8087	25.497	1	1	1	0.77421
CO (%)	Maximize	0.1093	0.3234	1	1	1	0.58686
HC (ppm)	Maximize	78.7325	156.563	1	1	1	0.60477
NOx (ppm)	Minimize	285.814	1069.53	1	1	5	0.63215
Smoke (N)	Minimize	1.47567	14.9431	1	1	5	0.80986

## Results and discussion

### Combustion characteristics

#### In-cylinder pressure

**Fig. 2:**
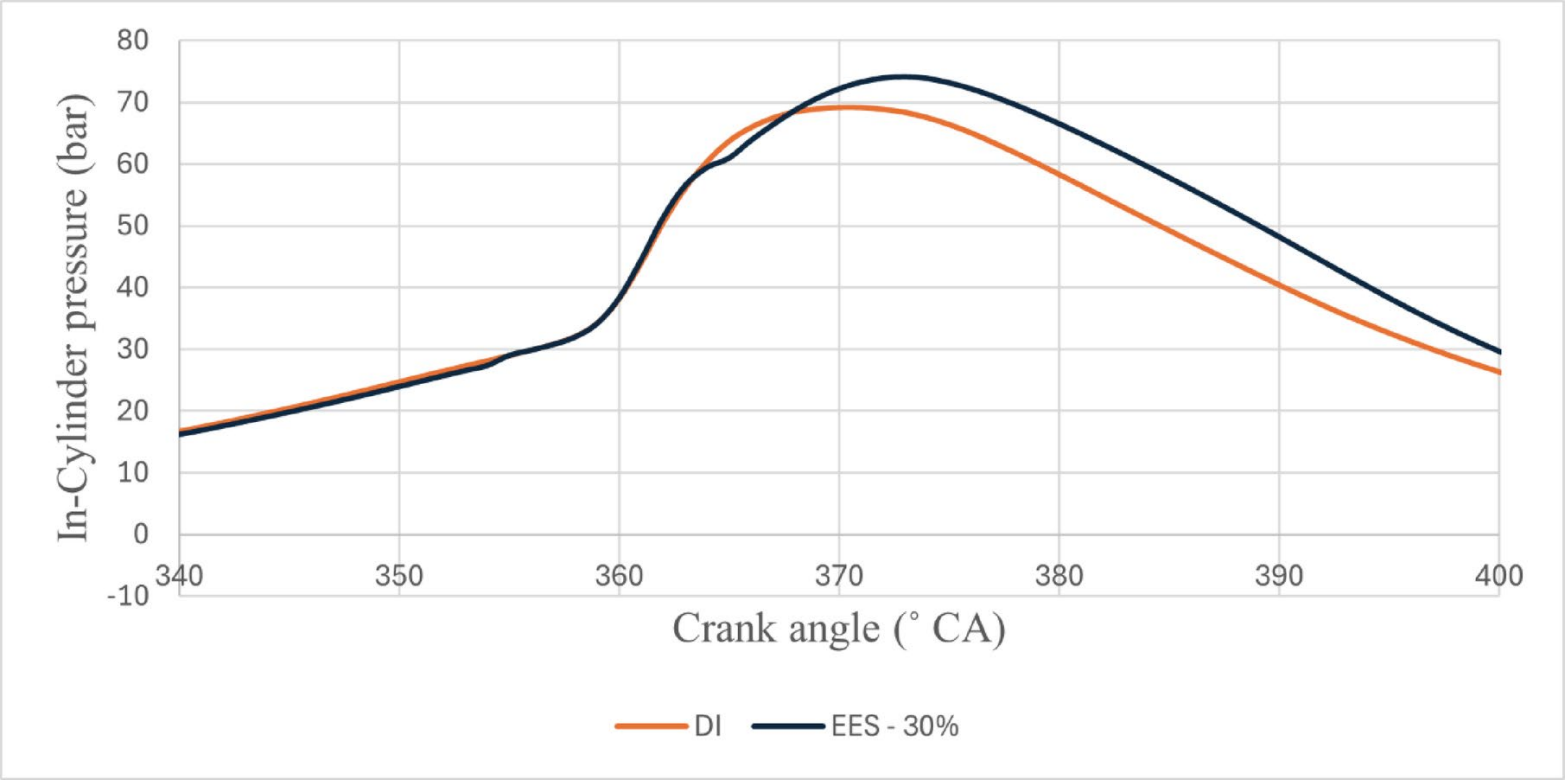
Variation of peak CP with EES at 80% load.

**Fig. 3:**
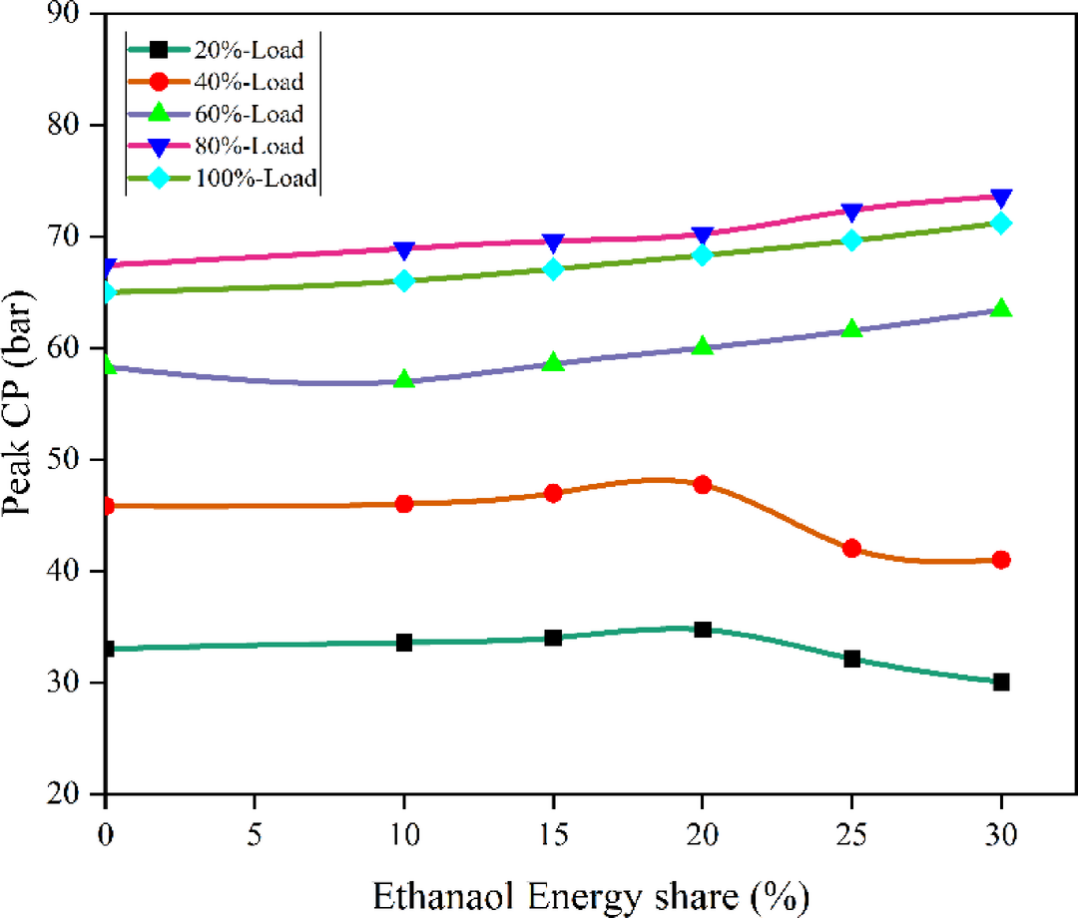
Variation of peak CP with EES at different loads.

The in-cylinder pressure is a measure of the quality of combustion inside the combustion chamber. As the fuel oxidises, it emits heat, and the gas mixture expands within the combustion chamber, increasing the cylinder pressure. Figures 2 and 3 - represents the peak cylinder pressure (CP) vs. EES, Peak CP values of RCCI mode of EES 10%, 15%, 20%, 25%, 30%, and M30 blend were 68.08, 69.85, 70.78, 72.51, 74.36, and 68.47 bar at 80% load respectively. The ID was lesser with the M30 blend, however. the M30 blend better atomization and vaporisation caused shorter ID due to biofuel chemical properties. Therefore, the M30 blend resulted in higher peak CP due to more premixed combustion. Higher fuel buildup during ID might be the result of fuel droplets that have been finely atomised using the advanced injection technique^42^.

**Fig. 4:**
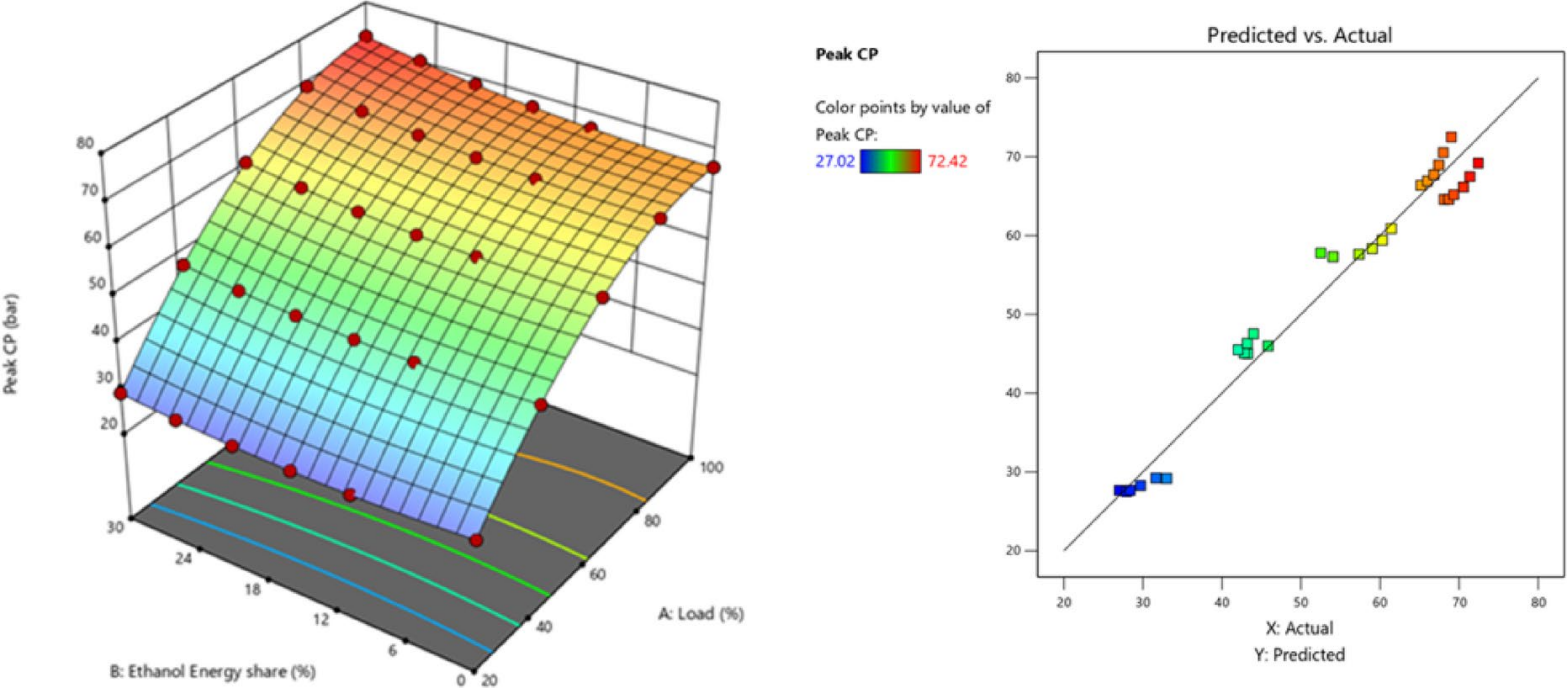
Optimization plot for peak CP with EES at different loads.

The peak CP traces of the ethanol RCCI mode are 8.6% higher than M30 blend. ON fuel injected into the intake port absorbed the heat produced by evaporation. As a consequence, the charge temperature and subsequent compression pressure increased. The intake charge (ethanol mixed with air) has a larger specific heat capacity than air, resulting in a higher in-cylinder temperature. Biodiesel combustion caused homogenous premixed ON fuel to burn simultaneously. While RCCI mode introduces ethanol into the intake manifold, where it evaporates before mixing with air, introduces into the cylinder, where it evaporates even more rapidly, leading to more O_2_ content of ethanol^29^. RCCI mode rich mixture in-cylinder charges raise temperatures and accelerate combustion. High LHV and ON fuels effect combustion, Singh et al. discovered. Due to faster air-fuel mixing, RCCI mode has richer regions. At higher load, peak CP outperformed RCCI. Alcohol injection via RCCI at the intake homogenises the cylinder mixture and starts the manifold air-fuel mixing process. Nevertheless, a concentration gradient of diesel combustion exists both within and outside the chamber. In the event of ethanol injection with PFI at the inlet, the manifold initiates the amalgamation of air and fuel, resulting in a more consistent composition within the cylinder. In contrast, a concentration gradient decreases from the interior of the chamber to the exterior with DI, as demonstrated in a study conducted by Li et al.^43^. Figure 4 shows the variation of predicted peak CP with respect to EES and engine load.

#### Heat release rate

**Fig. 5:**
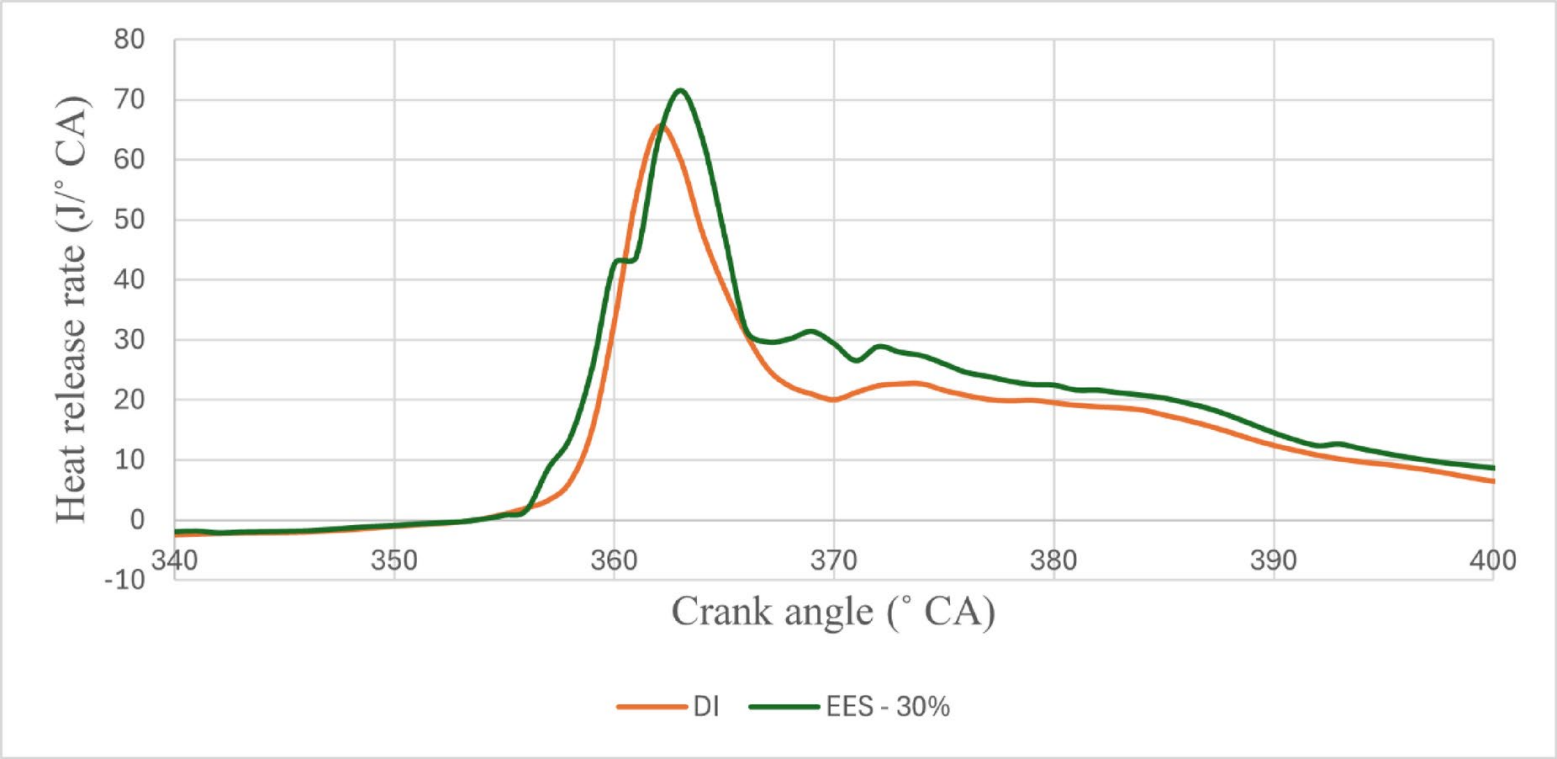
-Variation of HRR with EES at 80% load.

**Fig. 6:**
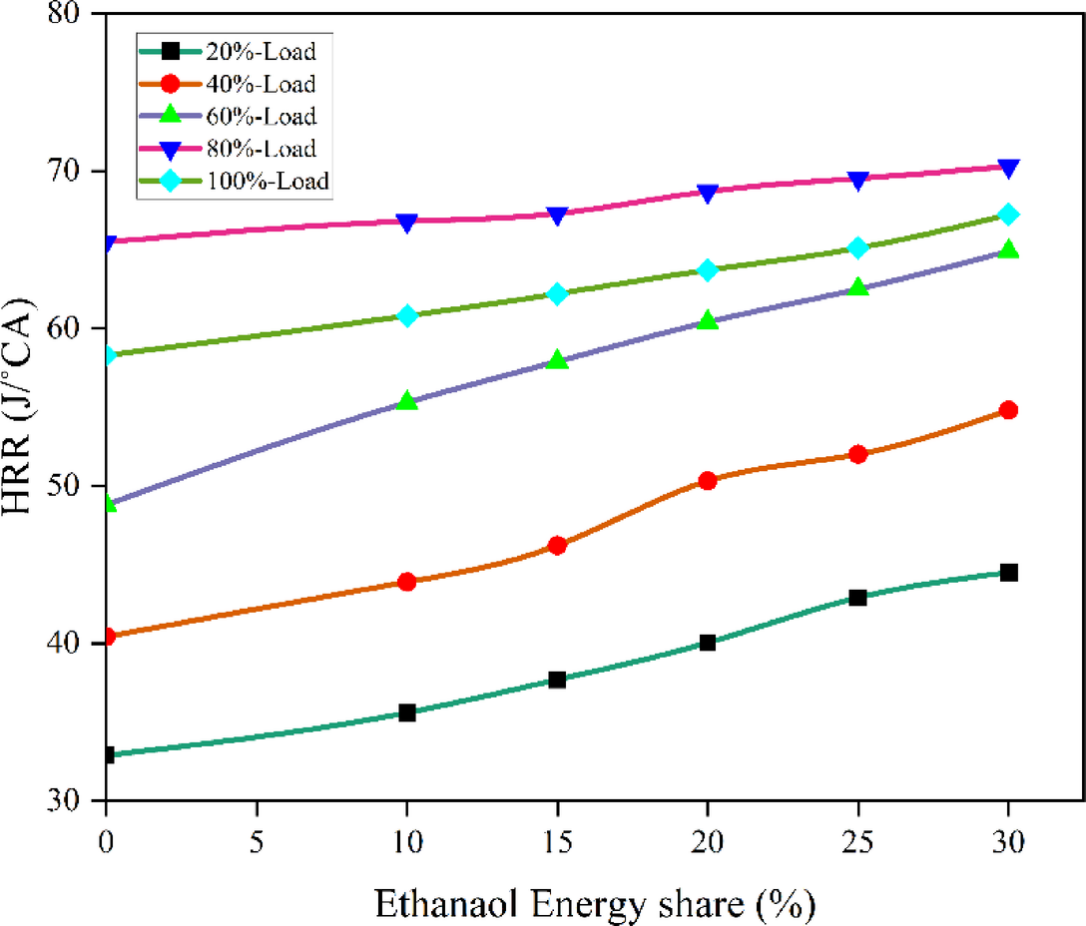
Variation of peak HRR with EES at different loads.

**Fig. 7:**
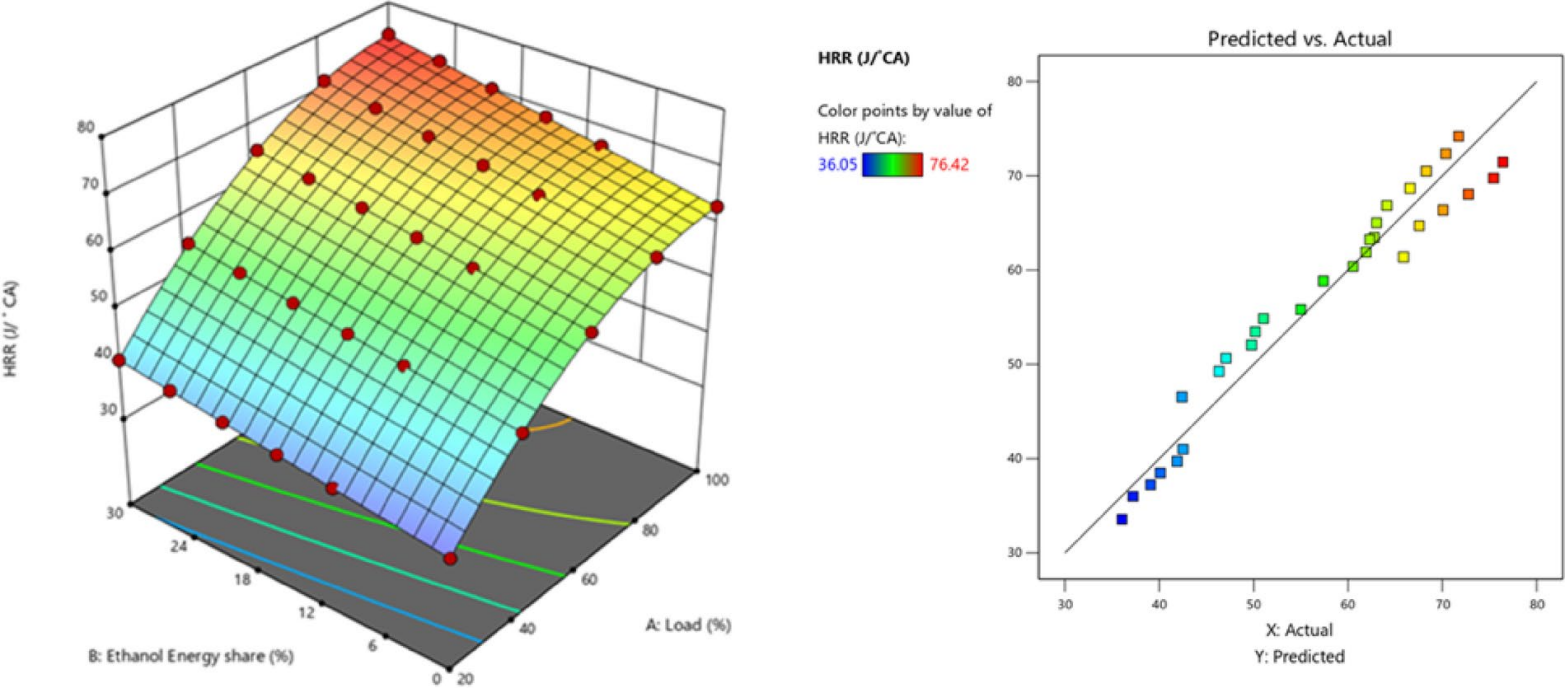
–Optimization plot for peak HRR with EES at different loads.

Single-stage premixed combustion is the result of the diesel atomising and evaporating prior to auto-ignition due to the extended ID period and reduced injection quantity of diesel under high load with RCCI strategy. Figures 5 and 6 - illustrates the peak HRR vs. EES, HRR values of RCCI mode of EES 10%, 15%, 20%, 25%, 30%, and M30 blend were 64.68, 65.79, 67.33, 68.05, 70.89, and 64.31 J/˚ CA at 80% load respectively. A significant quantity of reactive substances and active free radicals will build up in the combustion chamber, which reduces the inhibition effect of ethanol on the ignition of the M30 blend, makes it more likely that each point will be ignited at once, speeds up the combustion process oxidation and reaction rate, and shortens the ID of the RCCI. The M30 blend in RCCI mode will contribute more in diffusion combustion under the present injection conditions since the ID is greater than RCCI stage. The large amount of fuel burned increases the proportion of exhaust gas in the cylinder during DI mode, and this result decreases the instantaneous HRR, decreases the HRR, and prolongs the CD^44^. The HRR in RCCI mode 9.2% more than DI mode. RCCI mode injection of ethanol at the intake improves fuel-air mixture in the cylinder by mixing the two in the manifold. DI mode, on the other hand, creates an inside to outside descent-concentration gradient. HRR trends also revealed that RCCI mode, while diffusion phase combustion dominated in baseline DF combustion. In all two combustion modes, increasing engine load resulted in an increased peak HRR. As engine load increased, maximum heat release shifted to the bTDC, resulting in quicker fuel-air combustion kinetics^43^. Figure 7 shows the variation of peak predicted HRR with respect to EES and engine loads.

Diffusion combustion is less significant than premixed combustion in the RCCI mode with ethanol. When the mixture of ethanol and air is more uniform and has a higher laminar burning velocity, pre-premixed combustion occur.

RCCI mode further lesser CD even though it is already lower than RCCI. Because ethanol burns more quickly and has a larger O_2_ content, it enhanced the pace at which the air-fuel combination burned. In RCCI mode, the utilisation of M30 blend with a greater heating value than alcohol fuels is anticipated to result in in-cylinder temperatures exceeding those of DI M30 blend. Ningh et al., who reported same findings, indicated that ethanol RCCI allows for extended evaporation time and better air mixing in the manifold, resulting in a more homogenous charge inside the cylinder, hence enhancing combustion^22^. Due to the short ID duration and high CD, the DI of the M30 blend is mixed quite well before ignition happens. such that the CD (CA05 - CA90) is shortened and the in-cylinder combustion speed is increased. In contrast to a single injection strategy, the RCCI mode of ethanol causes the CA50 of a DF combustion to advance first and then delay, while the CA90 advances continuously and the CA50 gets sooner.

**Fig. 8:**
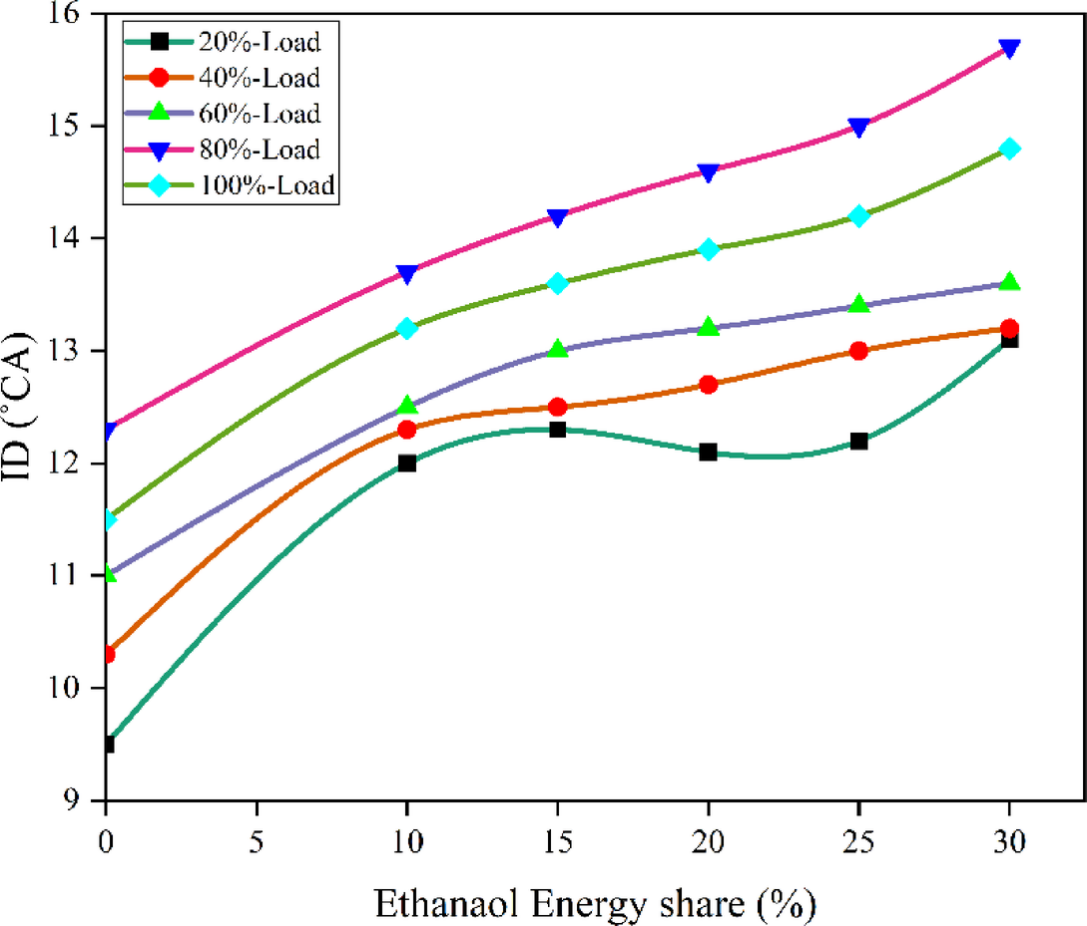
Variation of ID with EES at different loads.

**Fig. 9:**
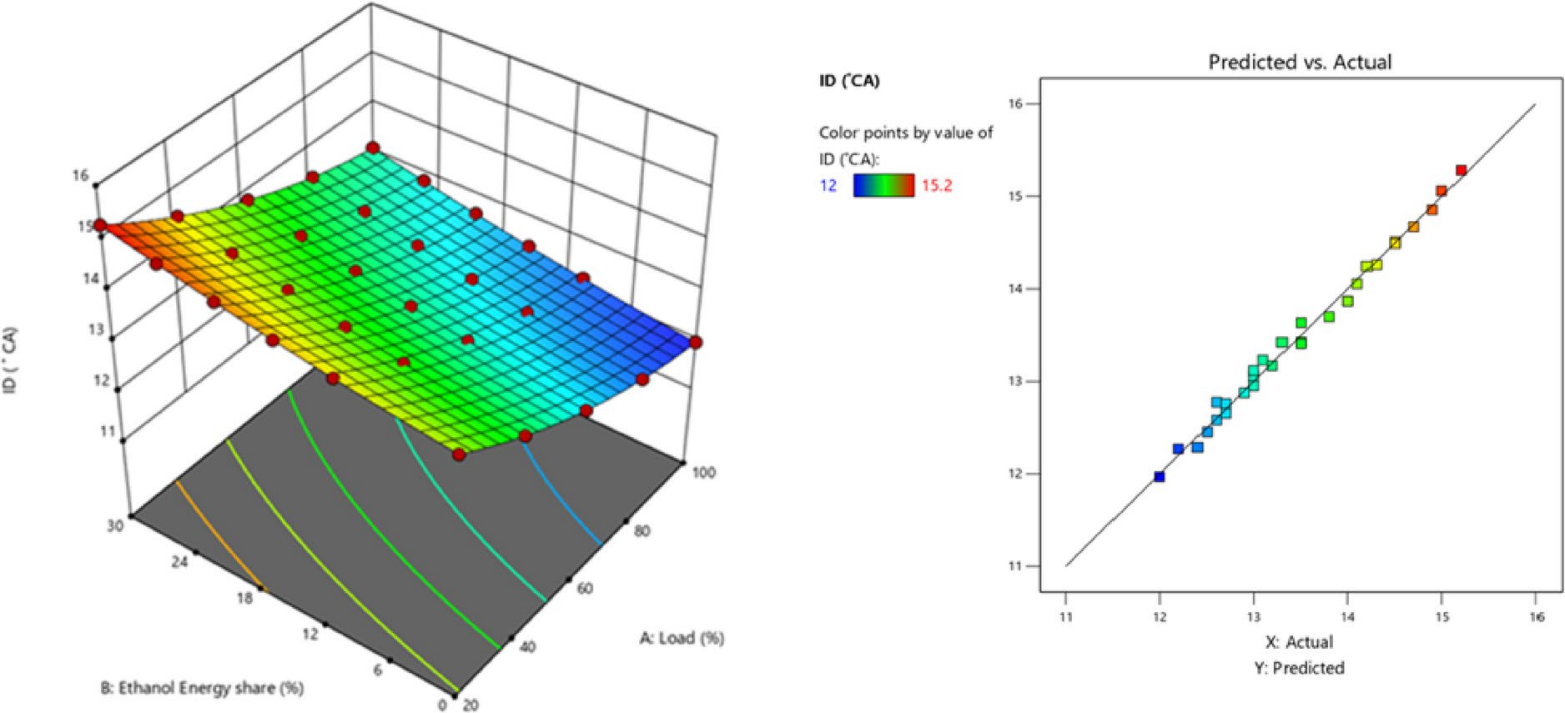
–Optimization plot for ID with EES at different loads.

Due to 10–30% of EES, the heat release in the first stage of RCCI mode is progressively delayed and diminished, leading to a postponement of CA05, but the primary HRR in the second stage is enhanced, causing CA50 to advance^45^. Figures 8 and 9 shows the variation of ID and predicted ID with respect to EES with engine loads. Ethanol absorbs more energy from compressed air than M30 blend because to its higher self-ignition temperature, ON, and LHV, which increases ignition time and delays combustion initiation. As a consequence, the ID grows in proportion to the mass flow rate of ethanol. The data show that when the bulk flow rate of ethanol rises, CD lowers. The highest quantity of fuel burns in the fast combustion phase aTDC in a low temperature environment, which results in a longer ID for homogeneous fuel. On the other hand, diffusion combustion uses relatively little fuel, which minimises CD. When using diesel RCCI engines with fuel consisting of alcohol, similar outcomes have been observed^46^.

**Fig. 10:**
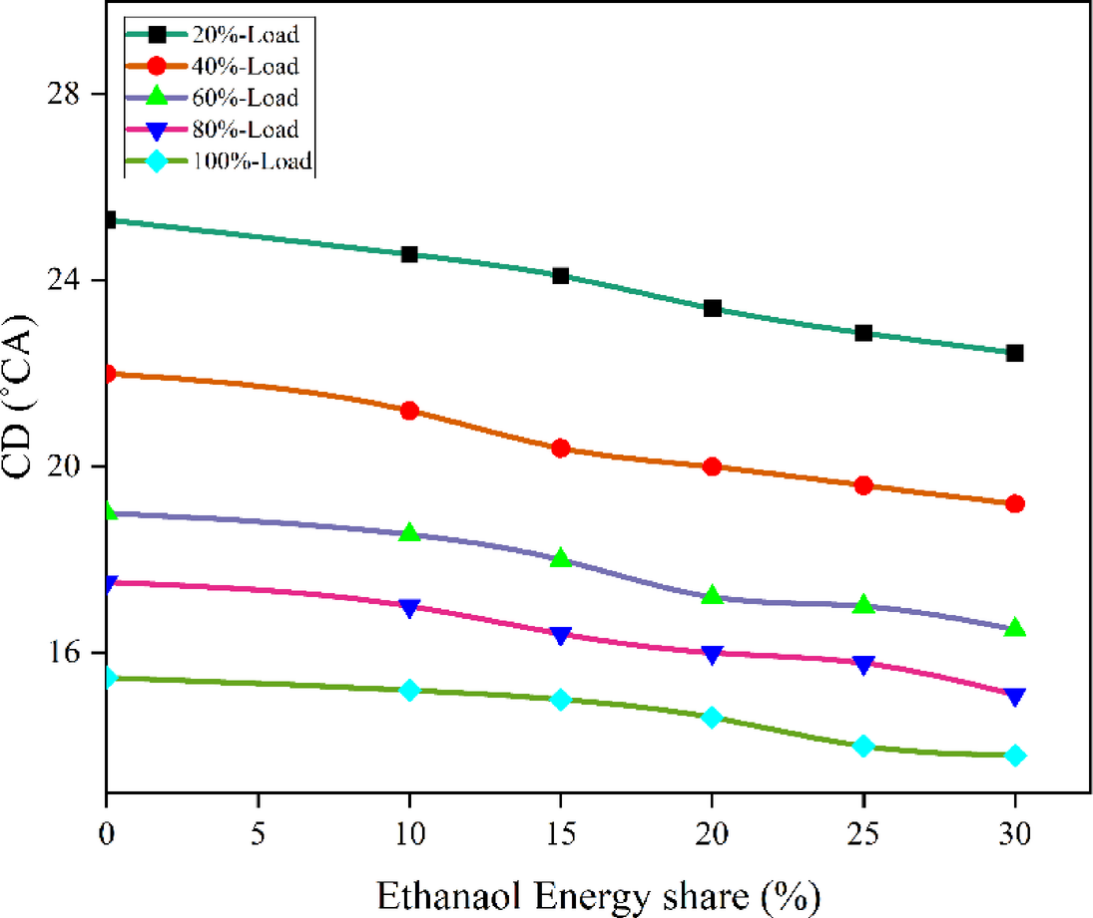
Variation of CD with EES at different loads.

**Fig. 11:**
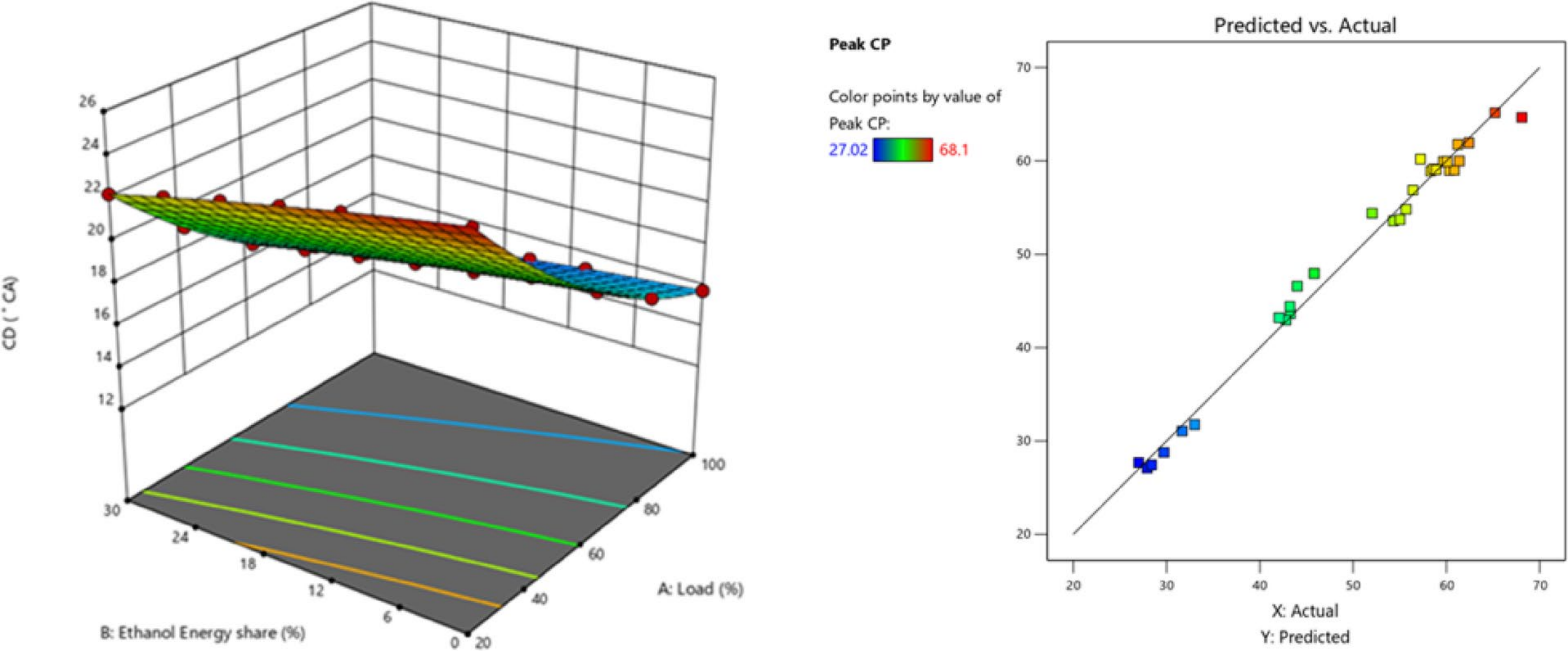
Optimization plot for CD with EES at different loads.

Due to the very low temperatures and pressures inside the cylinder, DI and combustion are entirely separated. As the cylinder temperature drops even lower before combustion with the addition of ethanol, the mixture’s reactivity drops as well, leading to longer mixing time, delayed combustion phasing, and burn length. Figures 10 and 11 shows the variation of CD and predicted CD with respect to EES with engine loads.

### Engine performance

#### Brake thermal efficiency

**Fig. 12:**
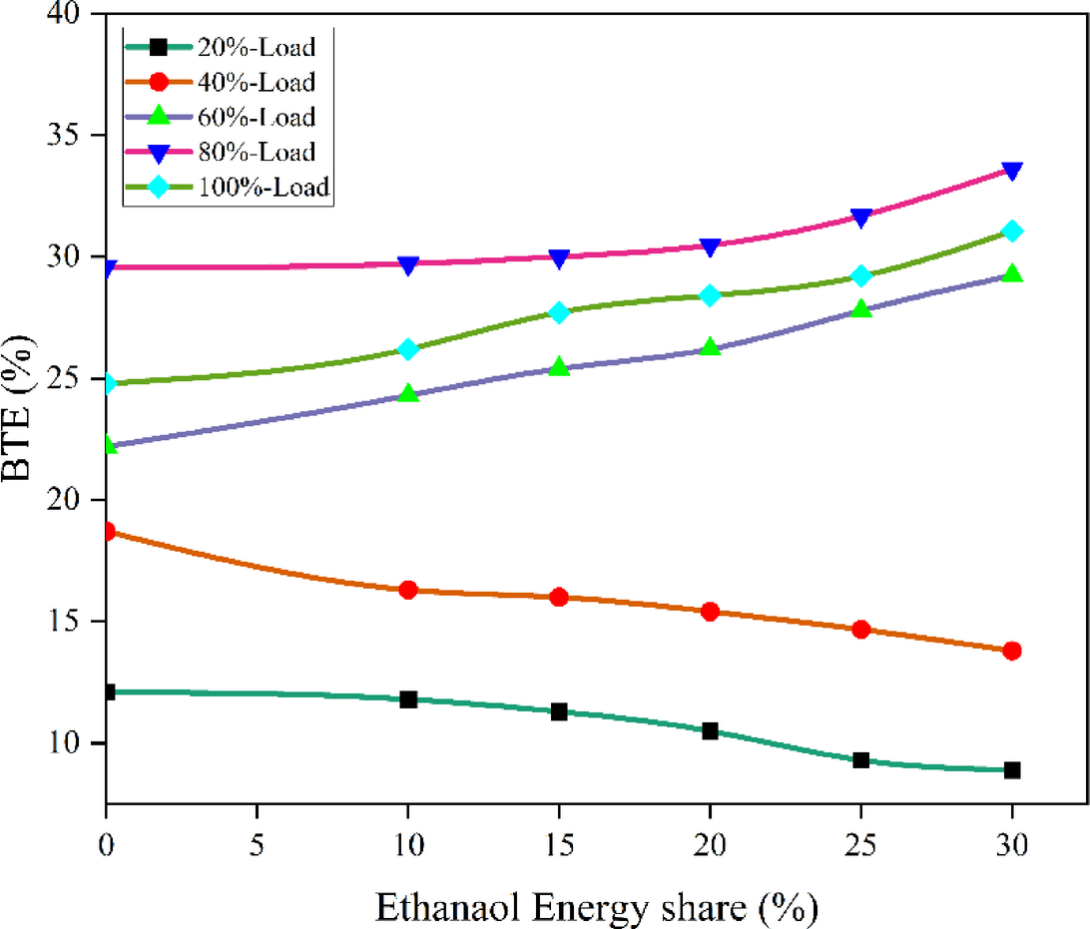
Variation of BTE with EES at different loads.

**Fig. 13:**
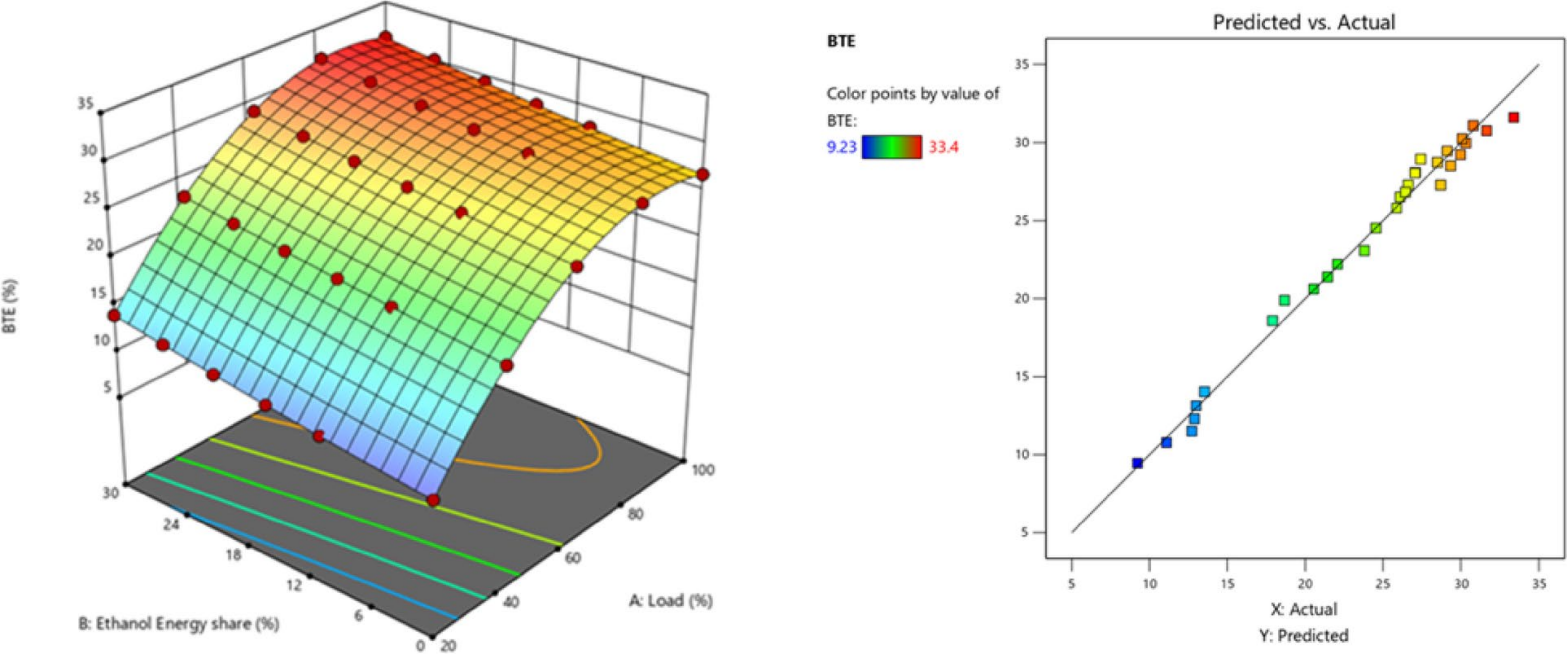
Optimization plot for BTE with EES at different loads.

BTE illustrates the engine’s efficacy in transforming the chemical energy of fuel into mechanical power. While the research in this article relies on constant engine operating circumstances, changes to the RCCI mode would influence combustion efficiency, hence influencing heat release and work capacity for the same fuel supply. Figure 12 - indicates the BTE at EES, BTE values of RCCI mode of EES 10%, 15%, 20%, 25%, 30%, and M30 blend were 29.71, 30.02, 30.47, 31.68, 33.6, and 29.5% at 80% load respectively. A higher BTE was achieved by the RCCI engine because to the enhanced cumulative charge’s CV caused by the PFI of ethanol into the engine via the intake manifold. Compared to diesel, the M30 blend produced higher BTE. The ignition duration increases with increasing ethanol concentration in RCCI mode, leading to the maximum CP and HRR around the TDC. Complete combustion and a more rapid release of peak heat were the outcomes of enhanced air-fuel mixing. The M30 blend lower CV than diesel resulted in a greater BSEC for the DF mode of EES^47,48^. The peak CP is virtually identical to that of a neat diesel engine, but owing to the uniform mixing of ethanol, total fuel is correctly used to generate sufficient power, hence BTE improves at 60%, 80%, and 100% engine load. The improvement in the homogeneity of the cylinder charge before combustion, the increase in constant volume combustion efficiency, and the higher O_2_ content in biodiesel were identified as factors contributing to the rise in BTE with ethanol and biodiesel. The main reason for the decline in the engine’s BTE with elevated energy share substitution (surpassing 30% EES) is the reduced reactivity of the DF mixture, resulting from the dominant charge dilution effect and the enhanced specific heat capacity of the charge^49^. M30 blend resulted in 12.2% lesser BTE than RCCI mode. Due to biodiesel contains more O_2_ level which leads to faster burning. An adequate amount of O_2_ is seen when HRF is used, lengthening the combustion phase and causing OH radicals to develop. Maximum HRR is achieved by using the better ignition capabilities of LRF and HRF to promote rapid flame propagation and combustion. Furthermore, the cylinder pressure smooth out due to the RCCI mode, showing continuous combustion. Figure 13 shows the variation of predicted BTE with respect to EES and engine loads.

#### Brake specific energy consumption

**Fig. 14:**
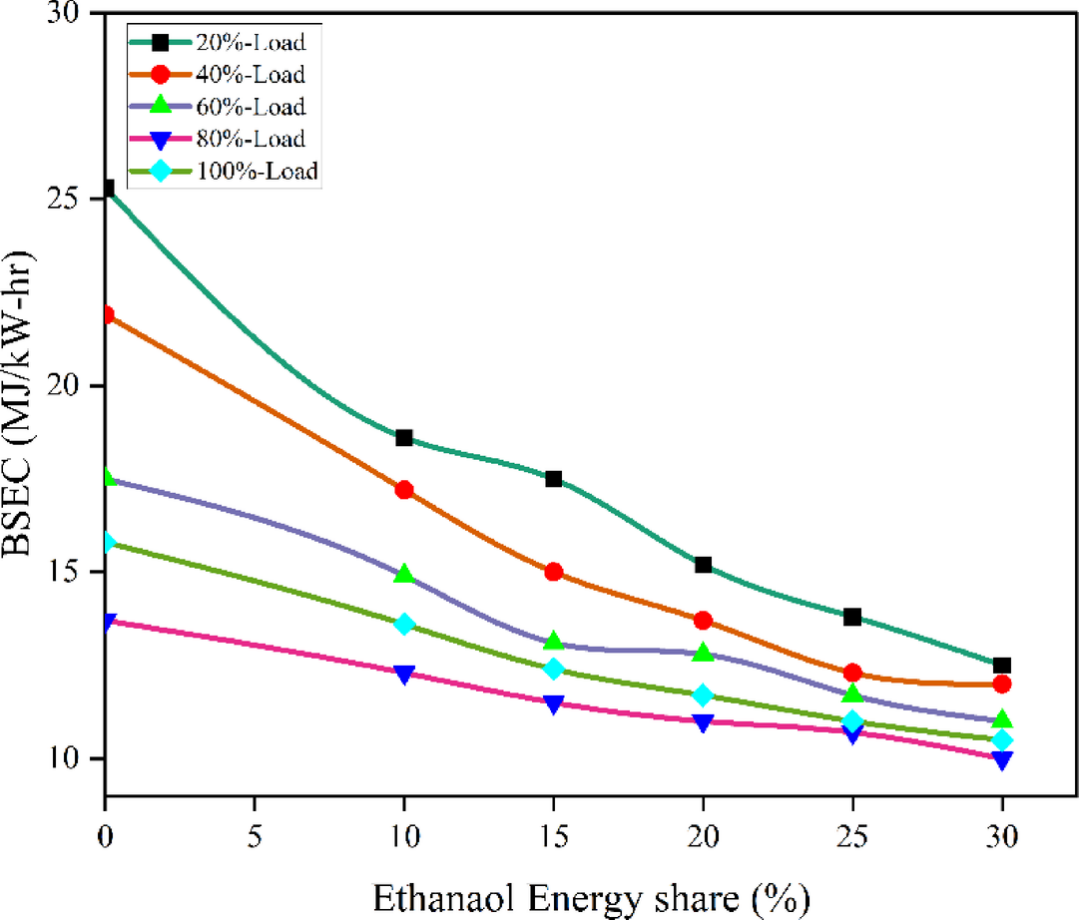
Variation of BSEC with EES at different loads.

Figure 14 demonstrate the BSEC values of RCCI mode. The BSEC for EES 10%, 15%, 20%, 25%, 30%, with M30 blend are 12.3, 11.6, 11.2, 10.9, 10.5, and 13.1 MJ/kWh at 80% load respectively. The RCCI combustion mode leads to improved reactivity distribution resulting in combustion efficiency and fuel consumption^50^. The oxygenated ethanol and biodiesel blend accelerate combustion, resulting in efficient fuel usage. As air inducted is replaced by ethanol vapour in the inlet, there is lack of free O_2_ in the cylinder to completely oxidise the fuel, resulting in more HC and CO.

**Fig. 15:**
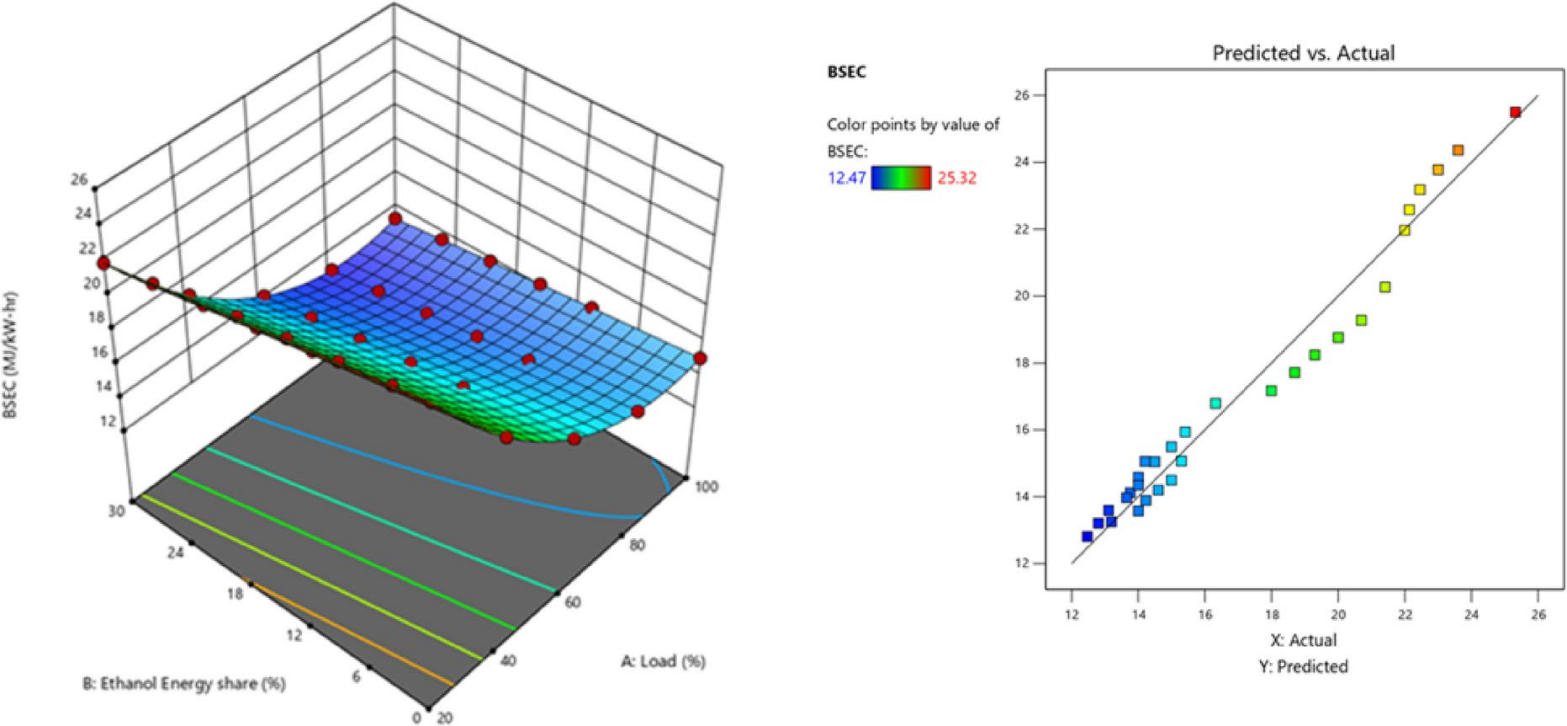
Optimization plot for BSEC with EES at different loads.

Because RCCI mode makes use of ethanol - which has a lower energy content than M30 blend the BSFC is somewhat lowered due to improved BTE compared to M30 in DI mode. Also, ethanol’s favourable mass-to-energy ratio reduces BSEC compared to M30 blend. The reduction in BSEC in RCCI mode can be ascribed to the increased O_2_ concentration resulting from the incorporation of ethanol fuel^51^. The RCCI mode showed higher BTE than DI mode of M30 blend at 60, 80, and 100% load. Since ethanol has less CV than M30 blend for the same power output requires more fuel, to make up for the low energy content resulting in higher BSFC but lower BSEC. Furthermore, the higher BTE of RCCI mode is a result of enhanced combustion efficiency caused by in-cylinder factors such high temperature at medium loads. At the same time, improved BTE is largely attributable to the fact that RCCI mode takes less time than DI mode, leading to lower heat transfer losses^29^. Figure 15 represents the variation of predicted BSEC with respect to EES and engine loads.

### Emissions characteristics

In summary, ethanol in RCCI mode leads to cleaner emissions in CI engines, while diesel offers higher energy efficiency but at the cost of higher emissions. This results already investigated by several authors vary in some respects and agree in others. While 68% report that CO have increased, almost 59% report NOx have decreased. There is a lot of coverage of these two occurrences in the literature since ethanol as a PFI in CI engines lowers combustion temperatures, which favours a decrease in NOx production and results in a greater LHV of ethanol. Usually caused by the high LHV, this temperature decrease contributes to the higher efficiency reported by 23% of the authors while the BSEC reported by 50% of them is decreased. These results suggest and encourage studies on the use of ethanol PFI in RCCI combustion. The in-cylinder temperature is lowered due to the high LHV of ethanol, but increased the quicker burning rates caused by the greater O_2_ concentration. Because ethanol fuel has more O_2_ available for burning, it produces more energy per unit of fuel, which raises the carbon content within the cylinder^52^. Because ethanol has low CN, increasing its proportion beyond certain limits decreases the cumulative CN. As a consequence, the ID is increased, causing charge to accumulate within the combustion chamber. As a consequence, a fuel-rich environment is generated within the combustion chamber, causing improper combustion and higher HC^53^. The alcohol and air mixture is homogenous due to its high volatility. Due to its low volatility, the injected M30 blend would stratify fuel vapour and air. The M30 blend vapour would have time to mix with the charge coarsely, creating a homogenous yet stratified combination. At several places, biodiesel vapour ignites the locally homogeneously mixed ethanol/air combination, yet the combustion is homogenous and flameless^54^. RCCI combustion had more HC and CO than DI.

**Fig. 16:**
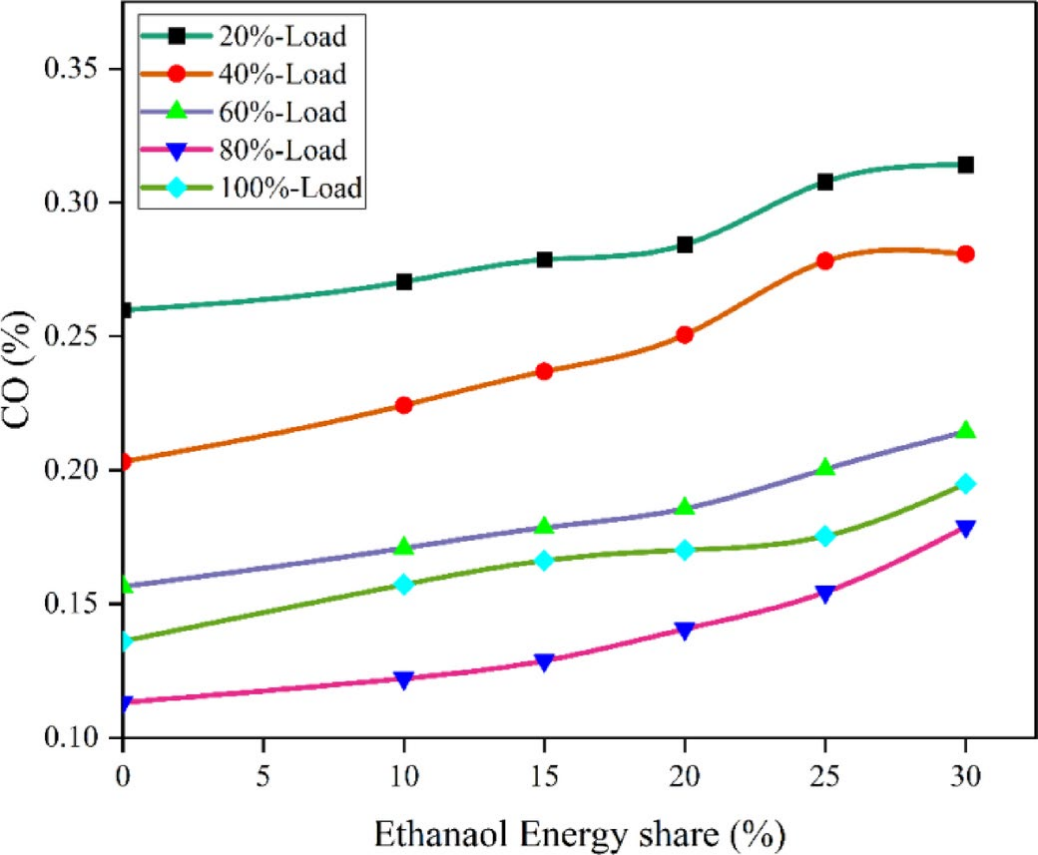
Variation of CO with EES at different loads.

**Fig. 17:**
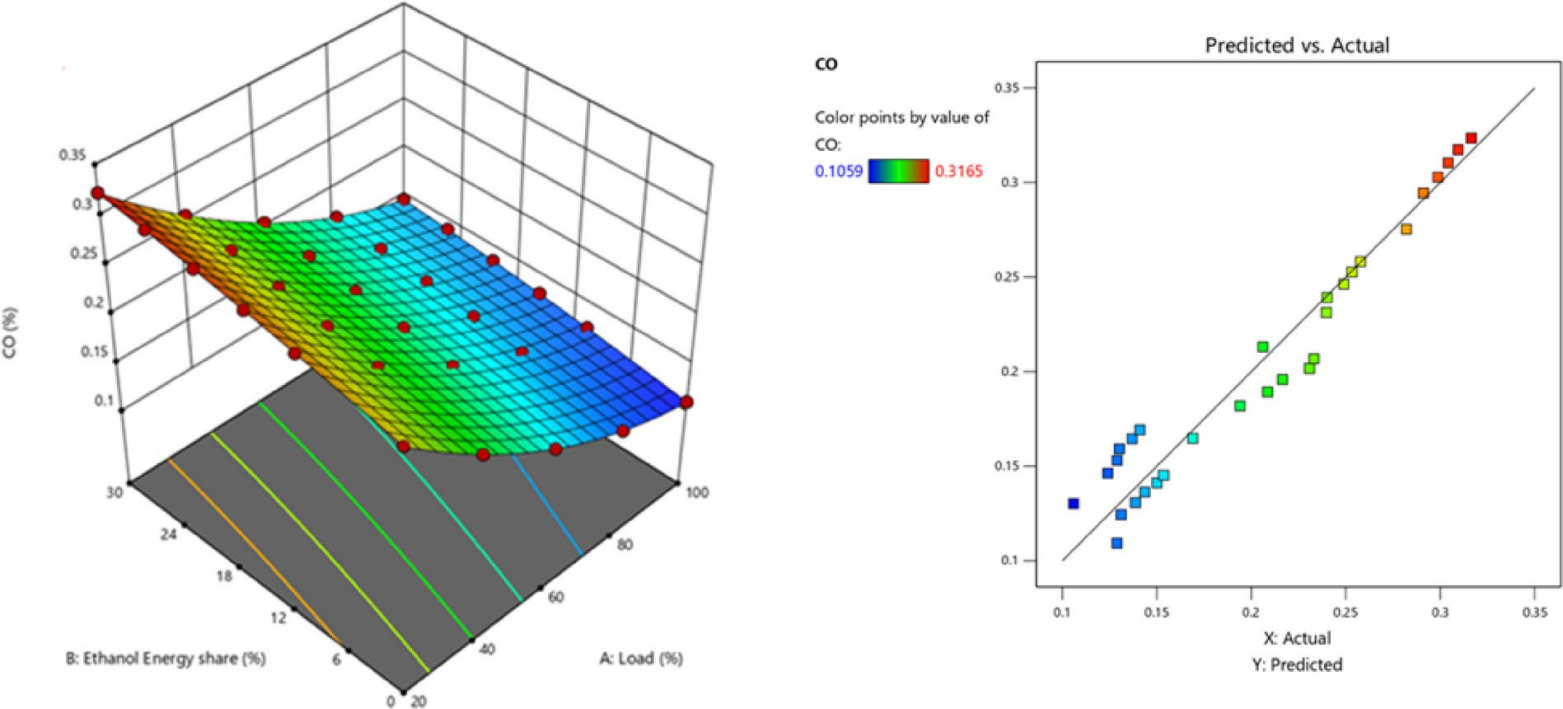
Optimization plot for CO with EES at different loads.

Higher EES indicates homogeneous ethanol. CO increases with homogeneous ethanol combustion cylinder temperature^55^. Ethanol premixed injection caused lean mixture and LTC, resulting in incomplete combustion. The longer ID from premixing allowed time to mix the combination, resulting in the lean mixture. Increased trapped ethanol adding into the splitting area also raises HC and CO. The premixed % of ethanol increased CO due to the rise in CA10 – CA50 and the reduction in rate of neat release rate(ROHR). Lower peak ROHR led lower global reaction rate, resulting in incomplete lean mixture oxidation^56^. The CO showed a pattern of steadily declining values after the addition of the RCCI of ethanol to the mixture. The overall increase in combustion allowed for higher cylinder temperatures, which in turn CO is converting CO to CO_2_^57^. Figure 16 - represents the CO at EES, CO values of RCCI mode of EES 10%, 15%, 20%, 25%, 30%, and M30 blend were 0.1222, 0.1359, 0.1406, 0.1545, 0.1780, and 0.1132% at 80% load respectively. Figure 17 indicates the variation of predicted CO with respect to EES and engine loads.

The RCCI mode CO 36.4% greater than DI. Nevertheless, when 30% of EES is present, the concentrations of CO and HC exceed the DI of the M30 blend. This is mostly attributable to the elevated % of ethanol entering the relatively low-temperature cutting down area, which is the principal source of HC. Furthermore, the CO is significantly influenced by subsequent oxidation. The increased EES with the M30 blend produced a homogenous mixture. At high loads, ethanol exhibits a greater LHV, resulting in cooling effect and oxidation of fuel mixture at reduced combustion temperatures. Under DF mode under part load, the concentration of the air/fuel mixture influences CO, among other parameters like as combustion temperature; the homogeneity of the air and fuel mixture leads to greater CO levels compared to DI mode^58^.

**Fig. 18:**
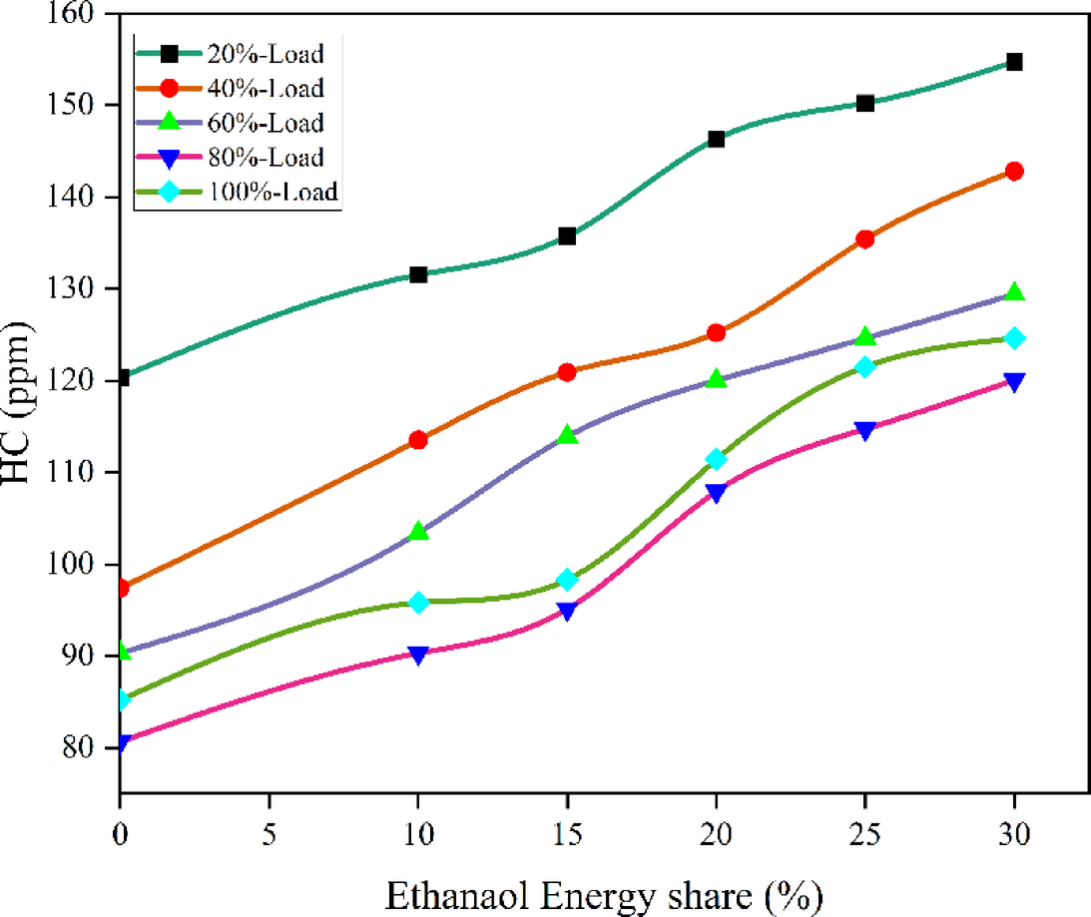
Variation of HC with EES at different loads.

**Fig. 19:**
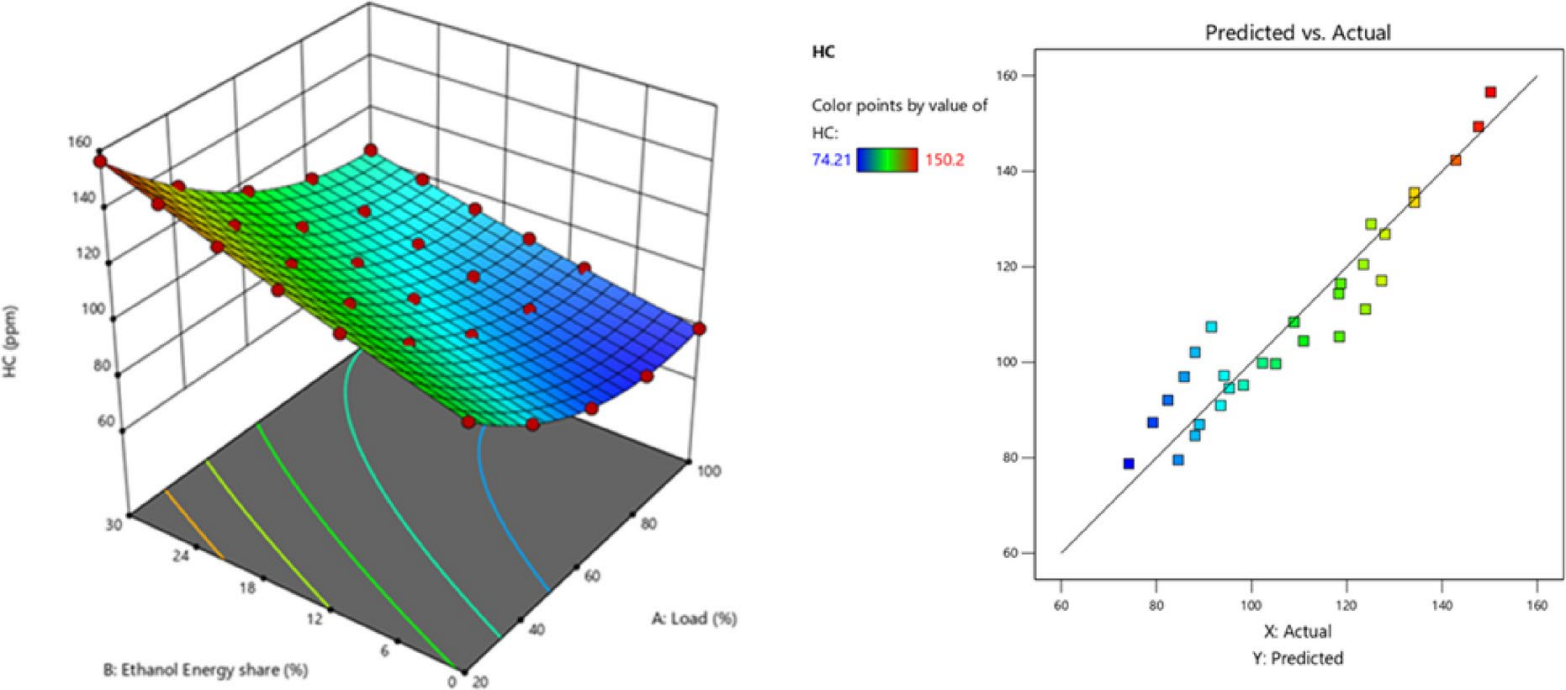
Optimization plot for HC with EES at different loads.

Research on DF mode engines utilising an ethanol-M30 blend similarly revealed an increase in HC and CO as ethanol content increased. Due to ethanol’s high evaporation temperature, the concentration of premixed ethanol rises, leading to increased levels of HC and CO. These happen when a CI engine is running in DF mode, which involves combustion at low temperatures and a greater fuel/air equivalence ratio^59^. Cold zones on the walls increased during compression, preventing fuel particles from completely combusting. Also, increasing combustion delay with ethanol gives fuel more time to reach crevice volumes and escape as HC. The RCCI mode of ethanol fuel delivers 34.9% higher HC than DI. Ethanol’s combustion tends to produce more HC, especially in homogeneous combustion conditions typical of DF mode. Figure 18 - illustrate the HC at EES, HC values of RCCI mode of EES 10%, 15%, 20%, 25%, 30%, and M30 blend were 90.65, 95.31, 107.72, 115.9, 123.9, and 80.6 ppm at 80% load respectively. Figure 19 demonstrate the variation of predicted HC with respect to EES and engine loads.

**Fig. 20:**
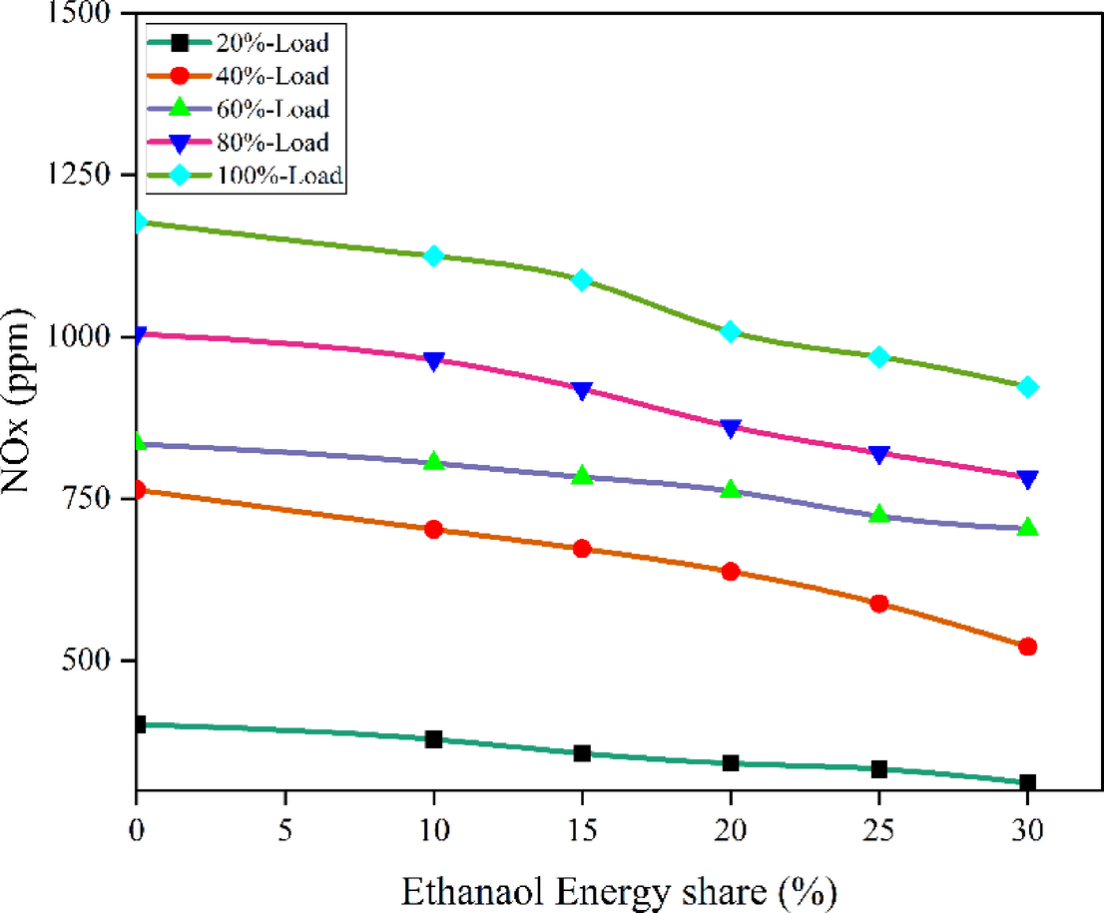
Variation of NOx with EES at different loads.

**Fig. 21:**
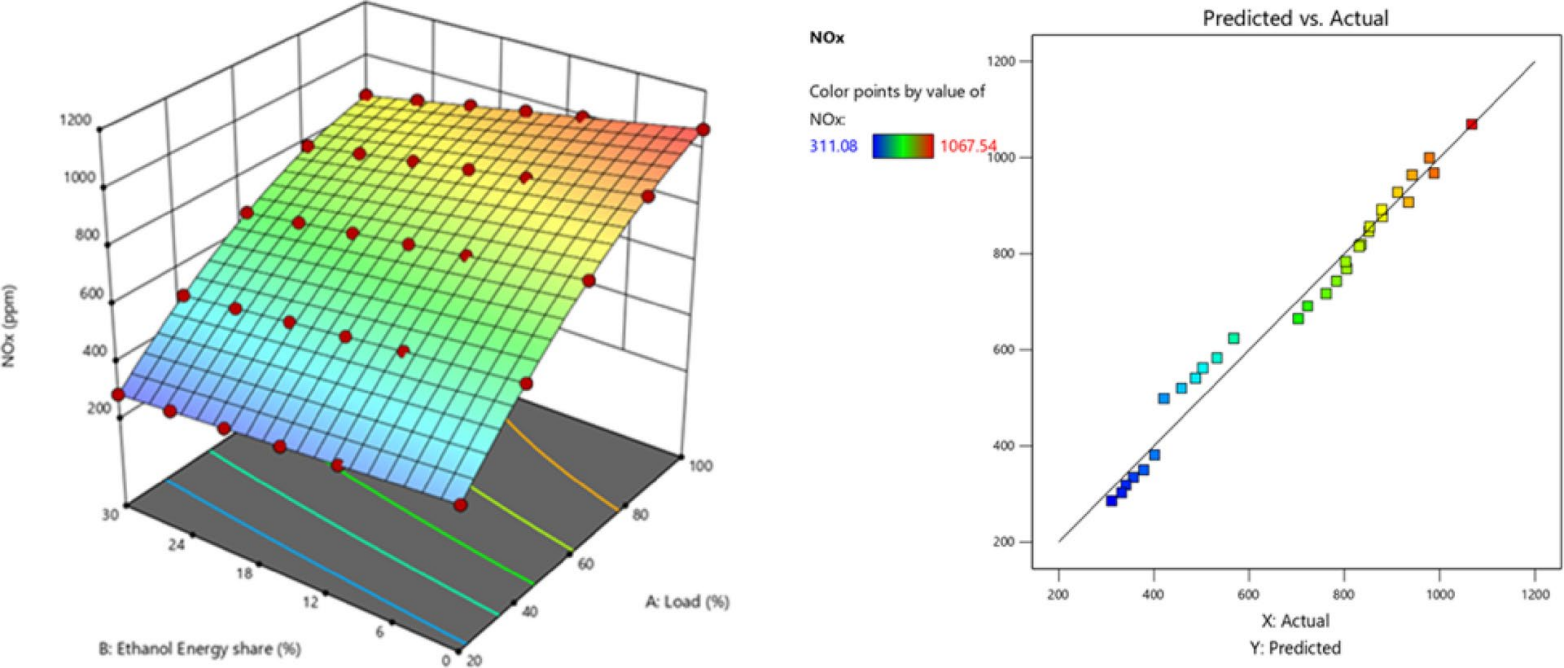
Optimization plot for NOx with EES at different loads.

NOx generation is largely affected by combustion temperature, O_2_ availability, and high temperature duration. Ethanol’s higher volatility and O_2_ content generally lead to lower peak combustion temperatures and more homogeneous combustion^60^. The PFI of the ethanol portion results in a decreased intake charge temperature, particularly at the conclusion of the compression phase, which provides a lower combustion temperature and subsequently reduces NOx production. In RCCI combustion mode, the air-fuel combination gets injected into the cylinder nearly uniformly. Compression triggers self-ignition everywhere. The cylinder is filled with a very lean, homogeneous mixture as a result of the intake and compression strokes. So, it is feasible to get high BTE while reducing NOx and smoke. Leaner mixtures are not a problem for RCCI engines. Though the bulk amount of HRF fed into the cylinder caused uncontrolled biodiesel combustion, increasing NOx the greater LHV of premixed ethanol lowers in-cylinder temperature. LTC reduces NOx and promotes RCCI combustion. Figure 20 - shows the NOx at EES, NOx values of RCCI mode of EES 10%, 15%, 20%, 25%, 30%, and M30 blend were 934.6, 870.12, 846.09, 823.11, 791.76, and 1023.41 ppm at 80% load respectively. Figure 21 shows the variation of predicted NOx with respect to EES and engine loads. The RCCI and DI combustion zone’s front had the 29.3% lowest NOx. For higher EES, a small HCCI zone within engine limits reduces NOx.

**Fig. 22:**
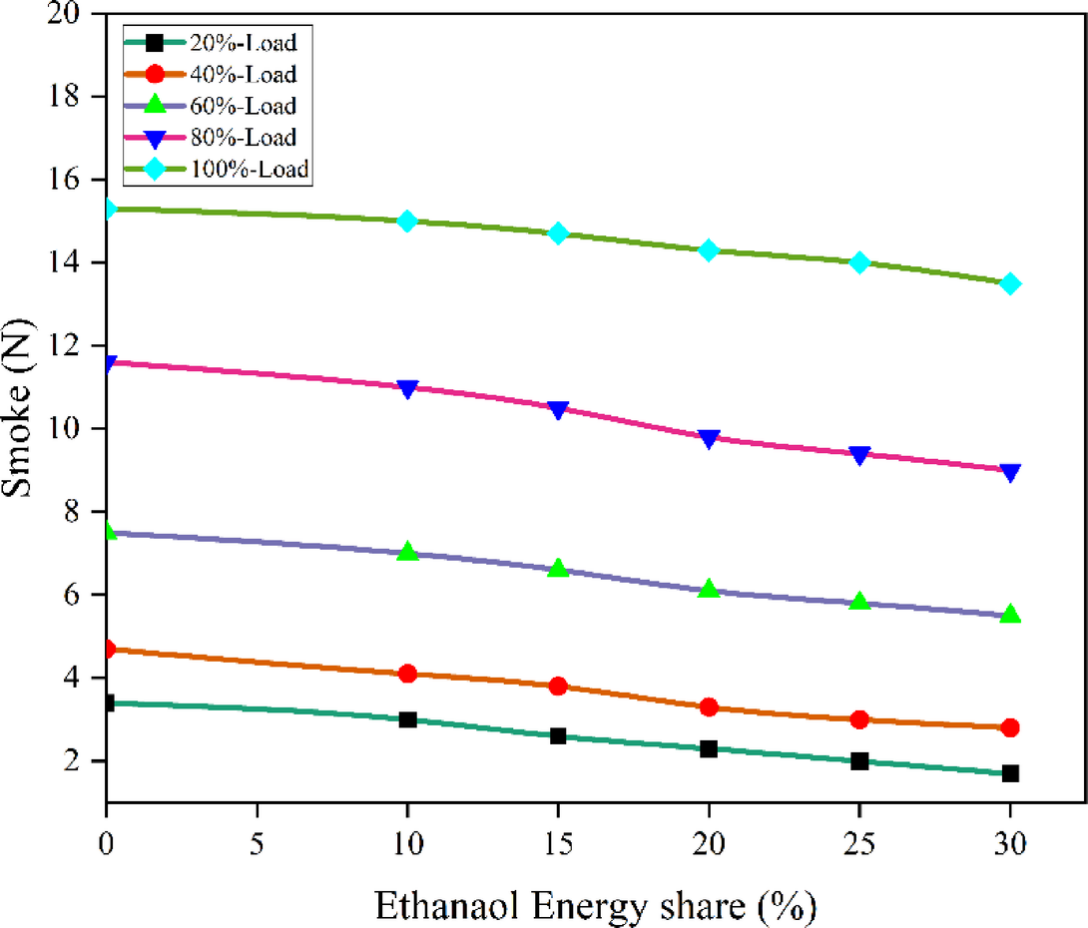
Variation of smoke with EES at different loads.

**Fig. 23:**
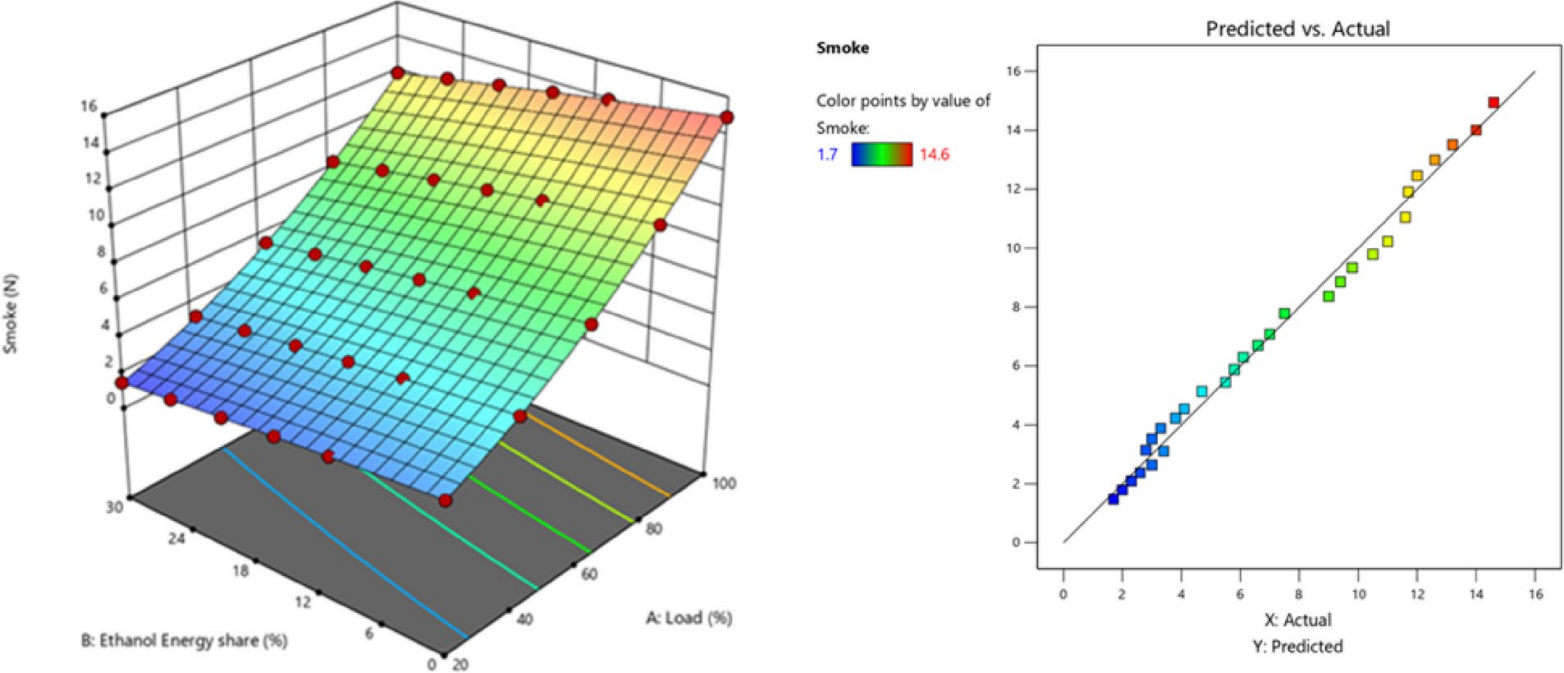
Optimization plot for smoke with EES at different loads.

Because of ethanol’s weaker C-C bond and LRF properties, combustion has led RCCI approaches to boost EES. This combustion method reduces smoke by raising peak temperature. Ethanol combustion does not create smoke like diesel and biodiesel. More lubricant was dissolved when EES impacted combustion chamber walls. Thus, more lubricant is degraded and burned. Only at high concentrations in energetic mode can ethanol-fired particulates peak because of lubricating oil. The RCCI combustion produces little smoke. As a straight-chain saturated monohydric alcohol with O_2_, ethanol is more likely to inhibit smoke-generated chemical pathways and minimise smoke^61^. Another reason for smoke formation is due to high fuel/air equivalence ratio and diesel fuel spray at intermediate temperatures^28^. Smoke is generated via partial oxidation, thermal cracking of fuel at elevated temperatures, and an excessively rich fuel combination. CO is the primary intermediate product generated during the combustion. Provided that the O_2_ concentration and temperature of the mixture in the cylinder are sufficiently elevated, and the duration of the chemical reaction is enough prolonged, CO will be oxidised to CO_2_^28^. Smoke and NOx exhibit a substantial trade-off connection in an RCCI mode, indicating that the combustion temperature in the cylinder is concurrently lower. However, with ethanol PFI smoke and NOx levels are lower^62^. Figure 22 - shows the smoke at engine load, smoke values of RCCI mode of EES 10%, 15%, 20%, 25%, 30%, and M30 blend were 11, 10.5, 9.8, 9.3, 8.7, and 11.6 N at 80% load respectively. The PFI mode of ethanol induction leads to 33.1% lesser smoke than DI mode. Figure 23 shows the variation of predicted smoke with respect to EES and engine loads.

## Optimization of engine variables

**Fig. 24:**
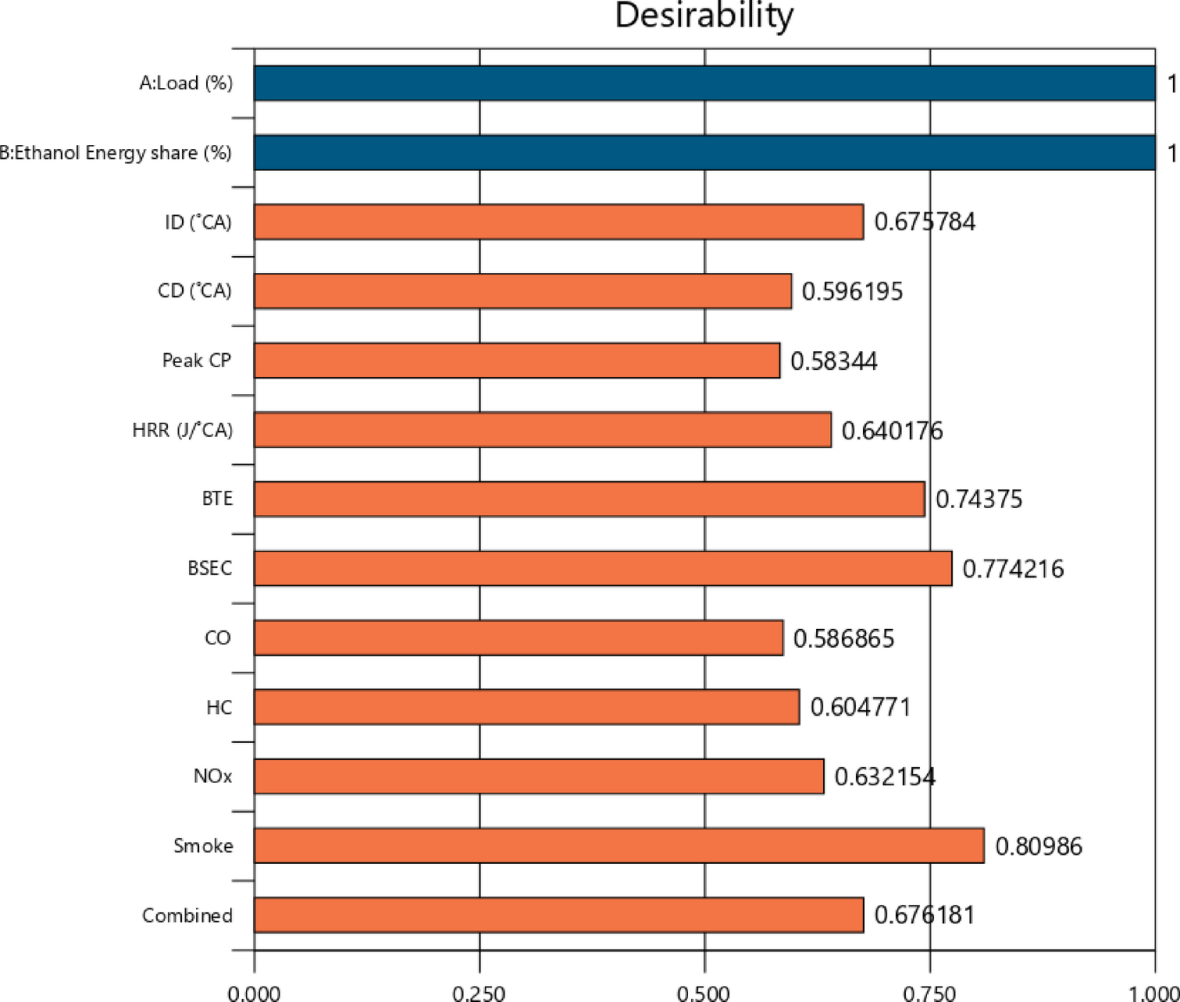
Desirability contour plot.

The optimization strategies aimed to enhance engine performance while simultaneously reducing BSEC and exhaust emissions, including NOx, HC, smoke, and CO. DOE-12 software was used in this study to optimise the influence of different injection strategy of M30 blend/ethanol fuel and various engine load conditions. This software optimized engine operating parameters based on the provided optimisation criteria, as well as input variables derived from these parameters. The model was created using the DOE - RSM optimiser module, which used a dimensionless desirability method with weightages ranging from 0 to 1 to evaluate each engine reaction. A answer with a minimum value of 0 is assessed unacceptable, whereas a response with a maximum value of 1 is considered ideal. The RCCI combustion of M30 blend/ethanol optimized engine predicted values of each output responses like, 72.68 bar, 68.44 J/˚ CA, 32.54%, 10.79 MJ/kWh, 0.1704%, 119.8 ppm, 8.35 N, and 764.08 ppm for peak CP, HRR, BTE, BSFC, CO, HC, smoke, and NOx, respectively at 83.4% of engine load and M30 blend with 28.43% of EES of RCCI combustion. Table 11 illustrates the engine parameters validation and weightage based desirability. The desirability technique is used to generate solutions based on the optimisation criteria. Figure 24 depicts the individual and combined desirability, which are determined by the nature of the quality attributes and the weighting factor. The desirability function technique converts expected responses into a dimensionless desirability score ranging from 0 to 1. A desirability score of 0 implies an undesirable response, while a score of 1 indicates that the response is the most desirable or perfect. To perform numerical optimisation on each target response, a particular criteria was specified. If the response falls outside of the accepted range, the desirability approaches 0. Depending on how the issue is presented, the purpose of each solution might be maximised, minimised, targeted, within a range, or equal to^63^.

**Table 11:** Validation test result and percentage of error with total desirability.

Parameters	Experimental data of RCCI mode combustion	RSM Prediction data	Error (%)
Peak CP (bar)	74.36	72.68	± 3.55
HRR (J/˚ CA)	70.89	68.44	± 3.33
BTE (%)	33.6	32.54	± 3.15
BSEC (MJ/kW-hr)	10.5	10.79	± 2.76
CO (%)	0.1780	0.1704	± 4.27
HC (ppm)	123.9	119.8	± 3.31
NOx (ppm)	791.76	764.08	± 3.55
Smoke (N)	8.71	8.35	± 4.6
	Over all desirability		67.62%

### Validation

Validation studies were carried out to confirm results. Validation studies were carried out using 30% biodiesel, varied EES, and engine loads to determine the best combination of input parameters. The trials were repeated three times to confirm that the results were reliable and reproducible. During the validation trials, the RSM models were evaluated for the ability to accurately forecast engine performance and emission characteristics under optimal circumstances. The experimental findings were compared to the expected values generated by the RSM models. Model accuracy was determined by computing the percentage error between experimental and predicted values. Validation trials demonstrated the durability and dependability of the created RSM models by their limited alignment with experimental data. The models can be effectively used within the experimental design framework to forecast engine performance and emissions. The validation studies confirm the accuracy of the optimisation method in determining the ideal combination of input parameters to get the required engine performance and emissions^64^.

## Conclusions

In this work, extensive engine testing were done across the entire load range to evaluate the performance, emissions, and fuel conversion efficiency of the PFI ethanol and DI mahua biodiesel-diesel fuelled LTC-RCCI combustion. The primary purpose of this work is to establish the optimal engine operating parameters and maximum ethanol substitution ratio that will result in optimal engine output using experimental study and RSM optimization. Quantitative comparisons provided a better knowledge of the potentials, requirements, and limitations of the ethanol-mahua biodiesel-diesel RCCI combustion.

The scripted conclusions from the established input parameters of 28.4% EES and 83.4% load are as follows:

- The BTE and HRR improved by 12.2% and 9.1% respectively.
- A significant smoke and NOx by 34.8% and 29.3% reduction is achieved with trade off to an increased HC and CO by 36.4% and 34.9%, but still within acceptable limits.
- The RSM output parameters of peak CP: 72.68 bar, HRR: 68.44 J/˚ CA, BTE: 32.54%, BSEC: 10.79 MJ/kWh, CO: 0.1704%, HC: 119.8 ppm, smoke: 8.31 N, and NOx: 764.08 ppm were realized.
- For the established operating conditions, the RSM predicted output values were validated with experimental data. The RSM predicted values compared with experimental results are within the acceptable range.

### Future study

Future research can include a thorough sensitivity analysis of the more input factors (low reactive fuel energy fraction, engine load, engine speed, and air–fuel ratio) to assess their impact on the output responses. Future research may broaden the established model to include other technical challenges, such as renewable energy and energy utilisation. Improvements may include improving energy fraction by using fuzzy theory in lieu of random values and utilising oppositional-based learning to enhance the effectiveness of the PRO algorithm in identifying optimum configurations. Simultaneous reduction of HC, CO, smoke and NOx can be researched.

## Data Availability

The datasets used and/or analysed during the current study available from the corresponding author on reasonable request.
